# The Transcription Factor *SUB1* Is a Master Regulator of the Macrophage TLR Response in Atherosclerosis

**DOI:** 10.1002/advs.202004162

**Published:** 2021-08-10

**Authors:** Rongzhong Huang, Zicheng Hu, Xiaorui Chen, Yu Cao, Hongrong Li, Hong Zhang, Yongyong Li, Liwen Liang, Yuxing Feng, Ying Wang, Wenhua Su, Zerui Kong, ND Melgiri, Lihong Jiang, Xingsheng Li, Jianlin Du, Yunqing Chen

**Affiliations:** ^1^ Department of Geriatric Medicine The Second Affiliated Hospital of Chongqing Medical University Chongqing 400010 China; ^2^ Institute of Ultrasound Imaging The Second Affiliated Hospital of Chongqing Medical University Chongqing 400010 China; ^3^ Department of Pulmonary and Critical Care Medicine The Second Affiliated Hospital of Chongqing Medical University Chongqing 400010 China; ^4^ Department of Cardiothoracic Surgery The First People's Hospital of Yunnan Province Kunming 650032 China; ^5^ Department of Cardiology The First People's Hospital of Yunnan Province Kunming 650032 China; ^6^ Department of Rehabilitation and Pain Medicine The Ninth People's Hospital of Chongqing Chongqing 400700 China; ^7^ Department of Rehabilitation Medicine The Second Affiliated Hospital of Chongqing Medical University Chongqing 400010 China; ^8^ Department of Cardiothoracic Surgery The Affiliated Yan An Hospital of Kunming Medical University Kunming 650000 China; ^9^ Yunnan Key Laboratory of Primate Biomedical Research Kunming 650500 China; ^10^ Impactys Foundation for Biomedical Research San Diego CA 92121 USA; ^11^ Department of Cardiology The Second Affiliated Hospital of Chongqing Medical University Chongqing 400010 China

**Keywords:** atherosclerosis, PC4, SUB1, TLR2, TLR4, Toll‐like receptor

## Abstract

Toll‐like receptor 2 and 4 (TLR2, TLR4) signaling is implicated in atherosclerotic plaque formation. The two‐stage master regulator Virtual Inference of Protein‐activity by Enriched Regulon (VIPER) analysis of macrophage TLR2 and TLR4 signature genes integrated with coexpression network genes derived from 371 patient‐derived carotid specimens identifies activated RNA polymerase II transcriptional coactivator p15 (*SUB1/Sub1*, *PC4*) as a master regulon in the atherogenic TLR response. It is found that TLR2 and TLR4 signaling is proinflammatory and proatherosclerotic in chow‐fed apolipoprotein E‐deficient (*ApoE* ^−/−^) mice. Through transgenic myeloid‐specific *Sub1* knockout in *ApoE* ^−/−^ mice, it is discovered that these proatherosclerotic effects of TLR2 and TLR4 signaling are mediated by Sub1. *Sub1* knockout in macrophages enhances anti‐inflammatory M2 macrophage polarization and cholesterol efflux. Irradiated low density lipoprotein receptor‐deficient (*Ldlr* ^−/−^) mice transplanted with *Sub1* ^−/−^ murine bone marrow display reduced atherosclerosis. Promoter analysis reveals Sub1‐dependent activation of interferon regulatory factor 1 (*Irf1*) transcription in a casein kinase 2 (Ck2)‐dependent manner, and *Sub1*‐knockout macrophages display decreased Irf1 expression. Artificial Irf1 overexpression in *Sub1*‐knockout macrophages enhances proinflammatory M1 skewing and lowers cholesterol clearance. In conclusion, the TLR master regulon Sub1, and its downstream effect on the transcription factor Irf1, promotes a proinflammatory M1 macrophage phenotype and enhances atherosclerotic burden in vivo.

## Graphical abstract

The Toll‐like receptor (TLR) master regulon RNA polymerase II transcriptional coactivator p15 (Sub1), and its downstream effect on the transcription factor interferon regulatory factor 1 (Irf1), promotes a proinflammatory M1 macrophage phenotype and enhances atherosclerotic burden in mice in vivo. The findings suggest that targeting the SUB1/IRF1 axis may be an effective strategy toward combating proatherogenic, TLR‐induced M1 polarization.

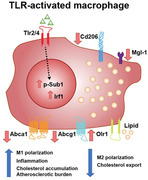

## 1 Introduction

Atherosclerosis is the leading cause of death and disability in developed countries and can precipitate a number of ischemic conditions, including coronary artery disease (CAD), carotid artery stenosis, ischemic stroke, peripheral arterial disease, and renal artery obstruction.^[^ ^1^ ^]^ Atherosclerosis is a disease caused by plaque build‐up within the cardiovascular system that results in a thickening of arterial walls, a decrease in arterial wall elasticity, and a narrowing of the arterial lumen. The progression of atherosclerotic plaque development is defined by vascular wall damage, subendothelial accumulation of oxidized low‐density lipoprotein (oxLDL), proliferation of fibrous connective tissue, and elevated recruitment of macrophages.^[^ ^2^ ^]^

The interaction between subendothelial oxLDL and macrophages is central to atherogenesis, as oxLDL induces macrophage inflammation and pathogenic foam cell differentiation in the vascular intima.^[^ ^3^ ^]^ This oxLDL‐induced macrophage activation is influenced by Toll‐like receptors (TLRs), a large family of membrane‐spanning, noncatalytic receptors that are displayed on the macrophage surface.^[^ ^4^ ^]^ Specifically, two TLRs—TLR2 and TLR4—have been linked to atherosclerotic plaque development. Both TLR2 and TLR4 contribute to foam cell accumulation within apolipoprotein E‐deficient (*ApoE* ^−/−^) murine aortic plaques, with TLR4 showing a more profound effect than TLR2.^[^ ^5^ ^]^ Moreover, TLR4 expression is upregulated within human atherosclerotic lesions and cultured macrophages exposed to oxLDL.^[^ ^6^ ^]^ Additionally, acute myocardial infarction patients display elevated TLR2 and TLR4 expression in circulating monocytes as well as elevated TLR4 expression in ruptured plaque macrophages.^[^ ^7^ ^]^ Therefore, macrophage TLR2 and TLR4 are considered potential therapeutic targets for atherosclerosis, and TLR2‐ and TLR4‐targeting agents are currently in preclinical development.^[^ ^8^ ^]^

Although macrophage TLR2 and TLR4 have well‐established roles in atherosclerotic plaque development, their downstream effectors and their roles in atherogenesis are not clearly understood. Here, we first sought to identify master regulator transcription factors (TFs) that determine the transcriptional response to atherogenic TLR2 and TLR4 stimuli. A two‐stage master regulator Virtual Inference of Protein‐activity by Enriched Regulon (VIPER) analysis was implemented to overlay macrophage TLR transcription signatures with six coexpression networks derived from 371 patient‐derived carotid specimens to identify intersection among genes within the macrophage TLR transcription signatures and the associated master regulons in carotid atherosclerotic plaques. This VIPER analysis revealed that the transcription factor activated RNA polymerase II transcriptional coactivator p15 (*SUB1/Sub1*, *PC4*) is a novel master regulator of TLR2 and TLR4 signaling in carotid plaque macrophages. Based on this in silico evidence, we sought to explore what impact macrophage *Sub1* activity has on the development of murine atherosclerosis. We discovered that the proatherosclerotic effects of TLR2 and TLR4 signaling in chow‐fed *ApoE* ^−/−^ mice are mediated by Sub1. We also found that *Sub1*‐knockout macrophages display an anti‐inflammatory M2 polarized phenotype and enhanced cholesterol efflux. Moreover, irradiated low density lipoprotein receptor‐deficient (*Ldlr* ^−/−^) mice transplanted with *Sub1* ^−/−^ bone marrow showed decreased atherosclerosis. The inflammatory phenotype of *Sub1*‐deficient macrophages is restored by artificial overexpression of proinflammatory interferon regulatory factor 1 (*Irf1*), thereby lowering their cholesterol efflux ability. The present study thus identifies *Sub1* as a master regulon of the atherogenic TLR response in macrophages.

## 2 Results

### 2.1 Identification of Master Regulator TFs in the Macrophage TLR Response

**Figure 1:**
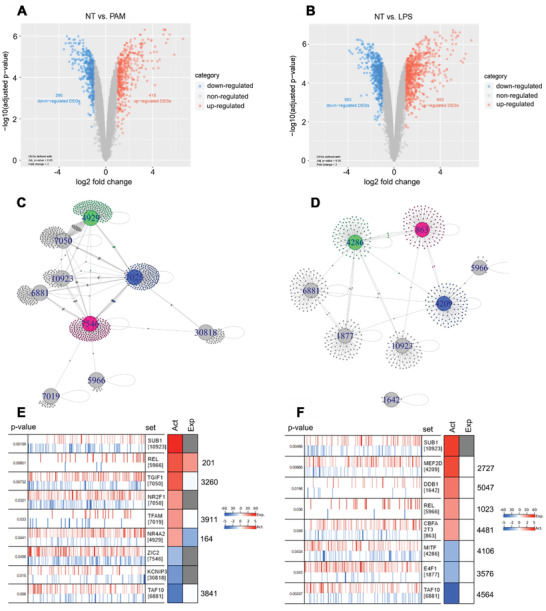
Identification of master transcription factors in the macrophage TLR2 and TLR4 responses. Differentially expressed genes (DEGs) identified between A) Pam‐treated (TLR2) and B) LPS‐treated (TLR4) versus nontreated bone marrow‐derived macrophages (BMDMs) represented by colored dots in a volcano plot; criteria for DEGs were: i) log_2_ fold‐change >1 and ii) *p* < 0.05; dots denote all transcripts detected by the microarray. Network diagrams of the master transcription factors (TFs) (large nodes labeled by TF's gene ID) and predicted targets (small dots) generated by Virtual Inference of Protein‐activity by Enriched Regulon (VIPER) analysis (*p* < 0.01) for C) the TLR2 network and D) the TLR4 network. Community structure predicted by fastgreedy.community module with edges denoting regulation of predicted targets by master TFs. VIPER evaluation of the master TFs for E) the TLR2 network and F) the TLR4 network. Right side: RTN and partial correlation analyses for repressed (blue) and activated (red) master TFs within coexpression networks reverse engineered and projected (vertical lines) to the TLR transcriptional signatures by msVIPER; *x*‐axis denotes the TLR signature sorted from most downregulated to most upregulated in Pam‐treated (TLR2) or LPS‐treated (TLR4) versus nontreated BMDMs as determined by the *t*‐statistic value from limma. Left side: The Act column of heatmap denotes activity, and the Exp column denotes value of differential expression within the TLR2 or TLR4 signature. Act signifies differential protein activity score in Pam‐treated (TLR2) or LPS‐treated (TLR4) versus nontreated BMDMs as predicted by the aREA module in VIPER, which analyzed coexpressed gene expression with TFs; red denotes increased activity, and blue denotes decreased activity post TLR2‐ or TLR4‐induction. Exp signifies differential transcript levels in Pam‐treated (TLR2) or LPS‐treated (TLR4) versus nontreated BMDMs as determined by the *t*‐statistic value from limma; red denotes upregulation, and blue denotes downregulation post TLR2‐ or TLR4‐induction.

TLR activation induces a restructuring of downstream signaling pathways, producing a pattern unique to TLR activity that enables identification of master regulator TFs that regulate this transcriptional program. By analyzing differences between the gene expression profiles of Pam_3_CSK_4_ (Pam)‐treated and lipopolysaccharide (LPS)‐treated versus nontreated bone marrow‐derived macrophages (BMDMs) (File S1, Supporting Information), we were able to extract the macrophage transcriptomic signatures associated with TLR2 and TLR4 activation, respectively. The differentially expressed genes (DEGs) identified in the BMDMs were mapped to orthologous human genes, and DEGs with no human counterpart were eliminated from further analysis. TLR2 activation was associated with 808 DEGs (418 upregulated and 390 downregulated genes; **Figure** **1**A), while TLR4 activation was associated with 1246 DEGs (683 upregulated and 563 downregulated genes; Figure 1B).

Next, we generated six coexpression networks from the six transcriptomic datasets derived from human carotid plaques and normal carotid tissue samples (*n* = 371) to compare to the TLR signatures in order to identify master regulator TFs that regulate the macrophage TLR transcriptional signatures in atherosclerosis. The four Affymetrix datasets (GSE21545, GSE24495, GSE43292, and GSE28829) were preprocessed before being combined to prevent possible bias, i.e., quality control (Figure S1, Supporting Information), normalization, and correction for batch effects of specimen subgroups (Figure S2, Supporting Information). The two non‐Affymetrix datasets (GSE13922 and GSE1000927) were not subject to preprocessing. Variation in sample sizes was accounted for by implementing a shrinkage estimate of partial correlations^[^ ^9^ ^]^ across TFs and their gene targets within every dataset; each edge within a network related to a significant partial correlation. The summary statistics for the six coexpression networks are provided in File S2 in the Supporting Information. We confirmed that there was concordance across all six datasets in the identified regulons, i.e., groups of genes regulated as a unit, generally by the same TF. *p*‐values were transformed to *Z*‐scores, and the six coexpression networks were combined into one network; edges from bigger studies were given more weight by applying Stouffer's method,^[^ ^10^ ^]^ which assigns a sample size‐dependent weighted significance to each edge. The resulting combined network possessed edges that were coherent across every dataset.

VIPER analysis was then implemented to overlay the macrophage TLR transcription signatures with the coexpression network to identify intersection among genes within the signatures and the associated master regulons.^[^ ^11^ ^]^ The VIPER analysis identified enrichment of TLR2 transcription genes in nine master regulator TFs (in order of most activated to most suppressed): *SUB1* (NCBI Gene ID: 10923), the NF‐kB subunit Avian Reticuloendotheliosis Proto‐Oncogene (*REL*) (ID: 5966), TGFB Induced Factor Homeobox 1 (*TGIF1*) (ID: 7050), Nuclear Receptor Subfamily 2 Group F Member 1 (*NR2F1*) (ID: 7025), Transcription Factor A, Mitochondrial (*TFAM*) (ID: 7019), Nuclear Receptor Subfamily 4 Group A Member 2 (*NR4A2*) (ID: 4929), Zic Family Member 2 (*ZIC2*) (ID: 7546), Potassium Voltage‐Gated Channel Interacting Protein 3 (*KCNIP3*) (ID: 30818), and TATA‐Box Binding Protein Associated Factor 10 (*TAF10*) (ID: 6881)) (Figure 1C,E). Likewise, VIPER analysis identified enrichment of TLR4 transcription genes in eight master regulator TFs (in order of most activated to most suppressed): *SUB1* (ID: 10923), Myocyte Enhancer Factor 2D (*MEF2D*) (ID: 4209), Damage Specific DNA Binding Protein 1 (*DDB1*) (ID: 1642), *REL* (ID: 5966), CBFA2/RUNX1 Partner Transcriptional Co‐Repressor 3 (*CBFA2T3*) (ID: 863), Melanocyte Inducing Transcription Factor (*MITF*) (ID: 4286), E4F Transcription Factor 1 (*E4F1*) (ID: 1877), and *TAF10* (ID: 6881) (Figure 1D,F). Analyzing the structure of the TLR2 gene network, we found that the *SUB1*, *TGIF1*, *NR2F1*, *NR4A2*, *ZIC2*, and *TAF10* regulons formed the network core, while the *KCNIP3*, *REL*, and *TFAM* regulons were more peripheral players (Figure 1C,E). Similarly, in the TLR4 gene network, *SUB1*, *MEF2D*, *CBFA2T3*, *MITF*, *E4F1*, and *TAF10* regulons formed the network core, while the *REL* and *DDB1* regulons were more peripheral players (Figure 1D,F). Gene set enrichment analysis (GSEA) was applied to the fastgreedy.community‐identified gene communities within the TLR2 gene network (*n* = 7 communities) and TLR4 gene network (*n* = 7 communities) to reveal the biological relevance of the pathways regulated by the master regulons (File S3, Supporting Information). Notably, the REL‐associated communities in both networks showed Gene Ontology molecular function (GO MF) enrichment for organic cyclic compound binding (GO MF: 0097159), heterocyclic compound binding (GO MF: 1901363), nucleic acid binding (GO MF: 0003676), and RNA polymerase II distal enhancer sequence‐specific binding (GO MF: 0003705).

Notably, there were three master regulons common to both TLR signatures: *SUB1*, *REL*, and *TAF10*. Among these three, the role of *REL* has already been well‐characterized in atherogenesis.^[^ ^12^ ^]^ However, to our knowledge, *SUB1* and *TAF10* are novel master regulator TFs that have not been previously linked to atherosclerosis. Considering *SUB1*’s strong activation status and its more central position in both TLR networks, we chose to pursue further investigation on the role of macrophage *SUB1* in atherosclerosis.

### 2.2 TLR2 and TLR4 Signaling Activates M1‐Skewing Sub1 in Murine Macrophages In Vitro and Atherosclerosis In Vivo

**Figure 2:**
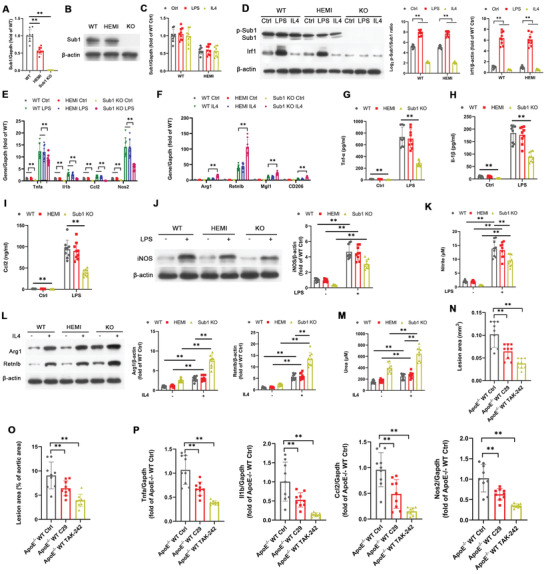
TLR2 and TLR4 signaling activates M1‐skewing Sub1 in murine macrophages in vitro and atherosclerosis in vivo. A–M) Bone marrow‐derived macrophages (BMDMs) isolated from *Sub1* ^flox/flox^ (wild‐type, WT), *LysM* ^Cre/−^/*Sub1* ^flox/wt^ (hemizygous, HEMI), and *LysM* ^Cre/−^/*Sub1* ^flox/flox^ (knockout, KO) mice were stimulated with vehicle (Ctrl), Pam (100 ng mL^−1^, 8 h), LPS (100 ng mL^−1^, 8 h), or IL4 (5 ng mL^−1^, 8 h). A) qPCR of *Sub1* mRNA levels. B) Immunoblotting of Sub1 protein levels. C) qPCR of *Sub1* mRNA expression. D) Immunoblotting of Sub1, p‐Sub1, and Sub1's downstream target Irf1. Densitometric quantification of the p‐Sub1/Sub1 ratio and Irf1 protein expression. E) qPCR of M1 marker genes. F) qPCR of M2 marker genes. ELISA of G) Tnf‐*α*, H) Il‐1*β*, and I) Ccl2 secretion. J) Immunoblotting of iNOS protein expression and K) nitrite‐based iNOS activity. L) Immunoblotting of Arg1 and Retnlb protein expression and M) urea‐based Arg1 activity. N–P) *ApoE* ^−/−^; *Sub1* ^flox/flox^ (*ApoE* ^−/−^ WT) mice were fed a chow diet and administered vehicle (Ctrl), C29 (50 mg kg^−1^), or TAK‐242 (3 mg kg^−1^) by daily intraperitoneal (i.p.) injection for 14 weeks. *n* = 9 mice per group. N) Quantification of aortic root lesion areas based on 8–12 10 µm sections per mouse (30 µm apart). Scale bar = 200 µm. O) Quantification of total lesion areas in en face aortas. Scale bar = 1 cm. P) qPCR of M1 marker genes in isolated aortic root plaque macrophages. Data reported as means ± SDs. In vivo experiments: *n* = 9 mice per group. In vitro experiments: *n* = 3 biological replicates × 3 technical replicates. *p < 0.05 and **p < 0.01 (A,O–P: one‐way ANOVA with Fisher's LSD; C–M: two‐way ANOVA with Fisher's LSD; comparing *n* = 3 in vitro biological replicates per group or *n* = 9 mice per group).

To understand the role of macrophage *Sub1* in TLR‐induced atherogenesis, we generated *Sub1* ^flox/flox^ (WT), myeloid‐specific hemizygous Lysozyme M (*LysM*)^Cre/−^/*Sub1* ^flox/wt^ (HEMI) mice, and myeloid‐specific *Sub1* KO mice (Figure S3, Supporting Information) and maintained BMDM cultures from WT, HEMI, and *Sub1* KO mice in vitro. Real‐time quantitative PCR analysis (qPCR) revealed Sub1 mRNA downregulation in HEMI mice and *Sub1* KO mice, with *Sub1* KO mice showing negligible levels of Sub1 mRNA expression (**Figure** **2**A). In contrast to mRNA levels, we observed similar Sub1 protein levels in WT and HEMI BMDMs (Figure 2B), suggesting haplosufficiency of *Sub1* (i.e., one WT allele is sufficient for normal protein expression). For all further experiments, both the WT and HEMI phenotypes were chosen as control groups to control for any *LysM* ^Cre^ transgene effects.

Macrophage phenotypes are polarized along a continuum from a proinflammatory M1 phenotype to an anti‐inflammatory, prorepair M2 phenotype.^[^ ^13^ ^]^ The TLR4 agonist LPS, and to a lesser degree the TLR2 agonist Pam, drive M1 polarization; conversely, the cytokine IL‐4 drives M2 polarization.^[^ ^14^ ^]^ As M1 macrophages promote atherogenesis while M2 macrophages promote atheroprotection,^[^ ^15^ ^]^ we applied these factors to drive BMDM polarization in vitro. Consistent with our in silico TLR analysis (Figure 1E), mRNA levels of Sub1 in primary BMDMs were not changed by Pam or LPS exposure (Figure 2C). However, the p‐Sub1/Sub1 ratio (i.e., an indicator of casein kinase II (CkII, Ck2)‐mediated Sub1 phosphorylation)^[^ ^16^ ^]^ and protein expression of Sub1's downstream target Irf1 were upregulated by Pam or LPS; the opposite effect was observed with the M2‐polarizing IL‐4 (Figure 2D). Decreased mRNA levels of the proinflammatory M1 markers Tumor Necrosis Factor Alpha (*Tnfa*, Tnf‐*α*), Interleukin 1 Beta (*Il1b*, Il‐1*β*), C‐C Motif Chemokine Ligand 2 (*Ccl2*, Ccl‐2), and Nitric Oxide Synthase 2 (*Nos2*, iNOS)^[^ ^17^ ^]^ were observed under vehicle, Pam, and LPS conditions in *Sub1* KO BMDMs (Figure 2E). However, higher mRNA levels of the anti‐inflammatory M2 markers Arginase 1 (*Arg1*, Arg1), Resistin Like Beta (*Retnlb*, Retnlb), Macrophage Galactose‐Type Lectin 1 (*Mgl1*, Mgl1), and CD206 Molecule (*CD206*, CD206)^[^ ^17^ ^]^ were observed under vehicle and IL‐4 conditions in *Sub1* KO BMDMs (Figure 2F). Secretion of the inflammatory markers Tnf‐*α*, Il‐1*β*, and Ccl‐2 was decreased in *Sub1* KO BMDMs under vehicle, Pam, and LPS conditions (Figure 2G–I). *Sub1* KO decreased iNOS protein expression and activity under vehicle, Pam, and LPS conditions (Figure 2J,K) but increased Arg1 and Retnlb protein expression as well as Arg1 activity under vehicle and IL‐4 conditions (Figure 2L,M). The above evidence confirms that TLR2 and TLR4 signaling activate Sub1 in murine macrophages, which skews them toward a proinflammatory M1 phenotype.

To validate the results of our findings in vivo, we conducted a series of experiments using pharmacological TLR inhibition or agonism in a chow‐fed *ApoE* ^−/−^ murine model of atherosclerosis. We administered the TLR2 inhibitor C29, the TLR4 inhibitor TAK‐242 (CLI‐095, resatorvid), or vehicle control to *ApoE* ^−/−^ mice fed a chow diet for 14 weeks. No significant differences in body weight or lipid profiles were observed between the three cohorts (Figure S4A–E, Supporting Information). Consistent with findings in chow‐fed *ApoE* ^−/−^ *Tlr2* ^−/−^ and *ApoE* ^−/−^ *Tlr4* ^−/−^ mice,^[^ ^5^ ^]^ C29 or TAK‐242 reduced aortic atherosclerotic burden (Figure 2N,O) and downregulated the proinflammatory M1 markers *Tnfa*, *Il1b*, *Ccl2*, and *Nos2* in macrophages isolated from aortic root plaques (Figure 2P). These findings confirm that TLR2 and TLR4 signaling are proinflammatory and proatherosclerotic in chow‐fed *ApoE* ^−/−^ mice.

### 2.3 Macrophage Sub1 Enhances TLR Signaling‐Induced Atherosclerosis in Chow‐Fed *ApoE* ^−/−^ Mice

**Figure 3:**
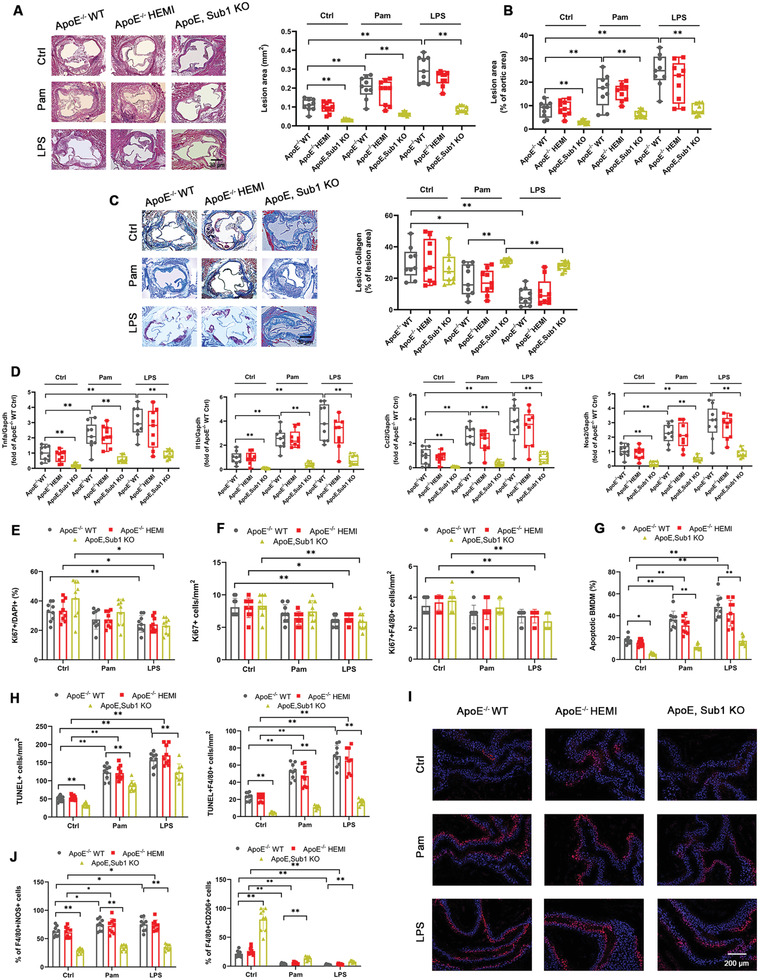
Macrophage Sub1 enhances TLR signaling‐induced atherosclerosis in chow‐fed *ApoE* ^−/−^ mice. *ApoE* ^−/−^; *Sub1* ^flox/flox^ (*ApoE* ^−/−^ WT), *ApoE* ^−/−^; *LysM* ^Cre/−^/*Sub1* ^flox/wt^ (*ApoE* ^−/−^ HEMI), and *ApoE* ^−/−^; *LysM* ^Cre/−^/*Sub1* ^flox/flox^ (*ApoE*, *Sub1* KO) mice were fed a chow diet and administered vehicle (Ctrl), Pam (15 µg), or LPS (50 µg) by weekly intraperitoneal (i.p.) injection for 14 weeks. A) Representative H&E staining images showing lesion areas in aortic root sections. Quantification of aortic root lesion areas based on 8–12 10 µm sections per mouse (30 µm apart). Scale bar = 200 µm. B) Quantification of total lesion areas in en face aortas. C) Representative Masson's trichrome staining images and quantification of aortic root lesion collagen by Zeiss Axiovision software. Scale bar = 200 µm. D) qPCR of M1 marker genes in isolated aortic root plaque macrophages. E) In vitro bone marrow‐derived macrophages (BMDMs) proliferation under vehicle (Ctrl), Pam (100 ng mL^−1^), or LPS (100 ng mL^−1^) for 8 h and F) in vivo F4/80+ macrophage proliferation in aortic root lesions examined using anti‐Ki67 immunofluorescence. Scale bar = 100 µm. G) In vitro BMDM apoptosis levels under vehicle (Ctrl), Pam (100 ng mL^−1^), or LPS (100 ng mL^−1^) for 8 h assessed by TUNEL staining. Cell morphology analyzed by differential interference contrast (DIC) and nuclear staining by DAPI. Apoptotic cell percentage expressed as ratio of TUNEL+/DAPI+. Scale bar = 100 µm. *n* = 9 fields per group. H,I) In vivo F4/80+ macrophage apoptosis in aortic root lesions examined by TUNEL and cleaved caspase‐3 staining. Scale bar = 100 µm. J) Immunofluorescent staining analysis of M1 macrophages (iNOS+/F4/80+) and M2 macrophages (CD206+/F4/80+) in serial aortic root lesion sections. Data reported as means ± SDs. In vivo experiments: *n* = 9 mice per group. In vitro experiments: *n* = 3 biological replicates × 3 technical replicates. *p < 0.05 and **p < 0.01 (two‐way ANOVA with Fisher's LSD; comparing *n* = 3 in vitro biological replicates per group or *n* = 9 mice per group).

To determine the role of Sub1 in TLR signaling‐induced atherosclerosis, we applied myeloid‐specific *Sub1* KO in the chow‐fed *ApoE* ^−/−^ murine model of atherosclerosis. We administered vehicle control, the TLR2 agonist Pam, or the TLR4 agonist LPS to *ApoE* ^−/−^; *Sub1* ^flox/flox^ mice (*ApoE* ^−/−^ WT), *ApoE* ^−/−^; *LysM* ^Cre/−^/*Sub1* ^flox/wt^ mice (*ApoE* ^−/−^ HEMI), and *ApoE* ^−/−^; *LysM* ^Cre/−^/*Sub1* ^flox/flox^ mice (*ApoE*, *Sub1* KO) fed a chow diet for 14 weeks. No significant differences in body weight were observed between the cohorts (Figure S5A, Supporting Information). Significant changes in lipid profiles were observed in LPS‐treated mice but not Pam‐treated mice; the LPS‐induced changes in total cholesterol, low‐density lipoprotein cholesterol (LDL‐C), and high‐density lipoprotein cholesterol (HDL‐C) were rescued by *ApoE*, *Sub1* KO (Figure S5B–E, Supporting Information). Pam or LPS enhanced aortic atherosclerotic burden and reduced lesion collagen deposition (as detected by Masson's trichrome stain) from *ApoE* ^−/−^ WT and HEMI mice, effects abrogated in *ApoE*, *Sub1* KO mice (**Figure** **3**A–C). Pam or LPS enhanced proinflammatory M1 marker expression in plaque macrophages from *ApoE* ^−/−^ WT and HEMI mice, effects abrogated in *ApoE*, *Sub1* KO mice (Figure 3D). As atherosclerotic progression is increased by macrophage proliferation within the atherosclerotic lesion,^[^ ^18^ ^]^ we analyzed macrophage cell proliferation and apoptosis in vitro and in vivo. LPS reduced, while *Sub1* KO had no significant impact, on macrophage proliferation in vitro or in atherosclerotic lesions in vivo by Ki67 immunofluorescence (Figure 3E,F). However, in vitro terminal deoxynucleotidyl transferase dUTP nick‐end labeling (TUNEL) assays showed that Pam or LPS enhanced apoptosis levels in macrophages in vitro or in atherosclerotic lesions in vivo, which were abrogated by *Sub1* KO (Figure 3G,H). Correspondingly, Pam or LPS enhanced cleaved caspase‐3 staining in atherosclerotic lesions in vivo, effects abrogated by *Sub1* KO (Figure 3I). Pam or LPS also enhanced iNOS+ M1 macrophage and decreased CD206+ M2 macrophage content in *ApoE* ^−/−^ WT and HEMI lesions, effects abrogated in *ApoE*, *Sub1* KO lesions (Figure 3J). These combined findings indicate that the proatherosclerotic effects of TLR2 and TLR4 signaling in chow‐fed *ApoE* ^−/−^ mice are mediated by Sub1.

### 2.4 Sub1 Mediates TLR Signaling‐Induced M1 Skewing in *ApoE* ^−/−^ Murine Macrophages

**Figure 4:**
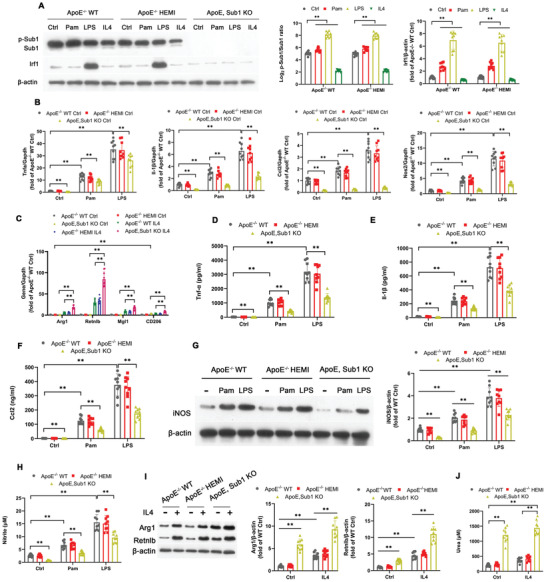
Sub1 mediates TLR signaling‐induced M1 skewing in *ApoE* ^−/−^ murine macrophages. Bone marrow‐derived macrophages (BMDMs) from *ApoE* ^−/−^; *Sub1* ^flox/flox^ (*ApoE* ^−/−^ WT), *ApoE* ^−/−^; *LysM* ^Cre/−^/*Sub1* ^flox/wt^ (*ApoE* ^−/−^ HEMI), and *ApoE* ^−/−^; *LysM* ^Cre/−^/*Sub1* ^flox/flox^ (*ApoE*, *Sub1* KO) mice were treated with vehicle (Ctrl), Pam (100 ng mL^−1^), LPS (100 ng mL^−1^), or IL4 (5 ng mL^−1^) for 8 h. A) Immunoblotting of Sub1, p‐Sub1, and Sub1's downstream target Irf1. Densitometric quantification of the p‐Sub1/Sub1 ratio and Irf1 protein expression. B) qPCR of M1 marker genes. C) qPCR of M2 marker genes. ELISA of D) Tnf‐*α*, E) Il‐1*β*, and F) Ccl2 secretion. G) Immunoblotting of iNOS protein expression and H) nitrite‐based iNOS activity. I) Immunoblotting of Arg1 and Retnlb protein expression and J) urea‐based Arg1 activity. Data reported as means ± SDs. *n* = 3 biological replicates × 3 technical replicates. *p < 0.05 and **p < 0.01 (two‐way ANOVA with Fisher's LSD; comparing *n* = 3 in vitro biological replicates per group).

We conducted a series of in vitro experiments utilizing Pam, LPS, and IL‐4 stimulation in BMDMs isolated from *ApoE* ^−/−^ WT, *ApoE* ^−/−^ HEMI, and *ApoE*, *Sub1* KO mice. Similar to WT BMDMs, the p‐Sub1/Sub1 ratio and protein expression of Sub1's downstream target Irf1 were upregulated by Pam or LPS; the opposite effect was observed with the M2‐polarizing cytokine IL‐4 (**Figure** **4**A). Pam or LPS upregulated the proinflammatory M1 markers *Tnfa*, *Il1b*, *Ccl2*, and *Nos2*, effects abrogated in *ApoE*, *Sub1* KO BMDMs (Figure 4B). The IL‐4‐induced anti‐inflammatory M2 markers *Arg1*, *Retnlb*, *Mgl1*, and *CD206* were upregulated in *ApoE*, *Sub1* KO BMDMs (Figure 4C). Pam or LPS upregulated secretion of the inflammatory markers Tnf‐*α*, Il‐1*β*, and Ccl‐2, effects abrogated in *ApoE*, *Sub1* KO BMDMs (Figure 4D–F). Moreover, Pam or LPS upregulated iNOS protein expression and activity, effects abrogated in *ApoE*, *Sub1* KO BMDMs (Figure 4G,H). IL‐4‐induced Arg1 and Retnlb protein expression as well as Arg1 activity were upregulated in *ApoE*, *Sub1* KO BMDMs (Figure 4I,J). This evidence reveals that proinflammatory M1 skewing by TLR2 and TLR4 signaling in *ApoE* ^−/−^ macrophages is mediated by Sub1.

### 2.5 oxLDL Exposure Activates Sub1, Which Reduces Cholesterol Efflux from Murine Macrophages

**Figure 5:**
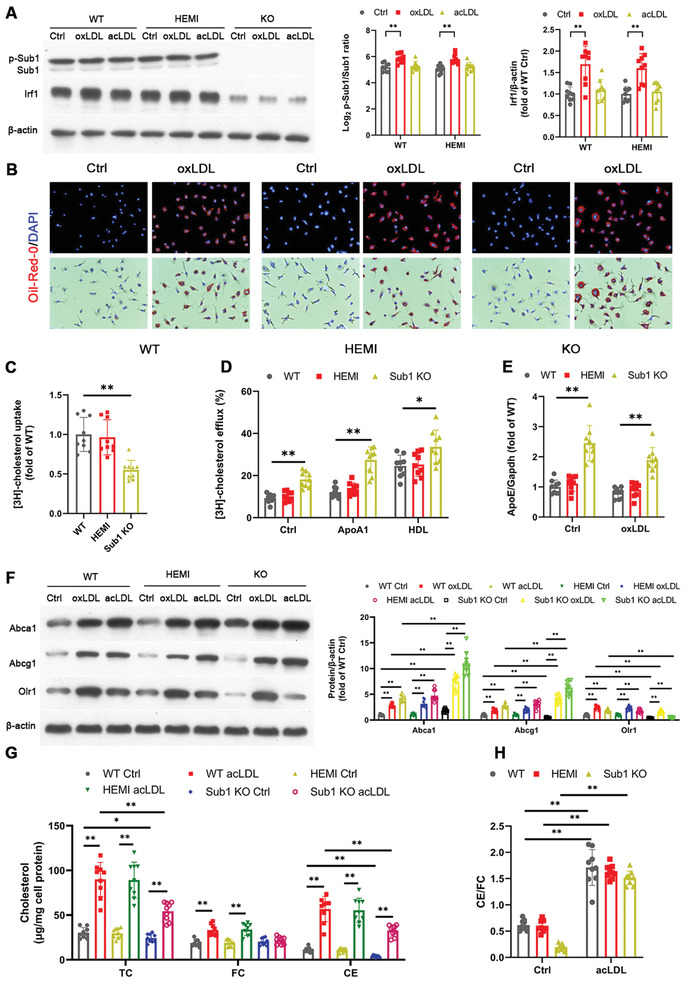
Macrophage Sub1 reduces cholesterol efflux from murine macrophages. The following experiments employed bone marrow‐derived macrophages (BMDMs) from *Sub1* ^flox/flox^ (wild‐type, WT), *LysM* ^Cre/−^/*Sub1* ^flox/wt^ (hemizygous, HEMI), and *LysM* ^Cre/−^/*Sub1* ^flox/flox^ (knockout, KO) mice. A) Immunoblotting of Sub1, p‐Sub1, and Sub1's downstream target Irf1 following exposure to vehicle (Ctrl) or modified LDLs (30 µg mL^−1^ oxLDL or 50 µg mL^−1^ acLDL, 24 h). Densitometric quantification of the p‐Sub1/Sub1 ratio and Irf1 protein expression. B) Representative images of neutral lipid accumulation upon oxLDL incubation (30 µg mL^−1^, 24 h). Scale bar, 100 µm. C) Cholesterol uptake after exposure to [^3^H] cholesterol‐labeled acLDL (50 µg mL^−1^) for 30 min. All values normalized to total protein levels and expressed as fold of WT. D) Cholesterol efflux after treatment with [^3^H] cholesterol for 48 h. E) qPCR of *ApoE* mRNA expression. F) Immunoblotting of Abca1, Abcg1, and Olr1 following exposure to Ctrl or modified LDLs (30 µg mL^−1^ oxLDL or 50 µg mL^−1^ acLDL, 24 h). G) Accumulation of total cholesterol (TC), free cholesterol (FC), and esterified cholesterol (CE), and H) the CE/FC ratio in BMDMs following acLDL exposure (50 µg mL^−1^, 24 h). Data reported as means ± SDs. *n* = 3 biological replicates × 3 technical replicates. *p < 0.05 and **p < 0.01 (A,E–H: two‐way ANOVA with Fisher's LSD; C,D: one‐way ANOVA with Fisher's LSD; comparing *n* = 3 in vitro biological replicates per group).

TLRs participate in the interaction between oxLDL and macrophages, thereby promoting oxLDL‐induced macrophage activation.^[^ ^3^, ^4^ ^]^ To understand the role of macrophage *Sub1* in oxLDL‐induced macrophage activation, we conducted a series of in vitro experiments in WT, HEMI, and *Sub1* KO BMDMs. We observed Sub1 activation in response to oxLDL exposure (**Figure** **5**A). After oxLDL exposure, we observed reduced lipid accumulation in *Sub1* KO BMDMs compared to WT and HEMI controls (Figure 5B). Moreover, lower [^3^H]‐cholesterol uptake (Figure 5C) as well as higher *ApoE*‐dependent autocrine cholesterol efflux (Ctrl; Figure 5C) and cholesterol efflux to Apolipoprotein A1 (ApoA1) and HDL‐C (Figure 5D) were observed in *Sub1* KO BMDMs. *ApoE* mRNA levels were also upregulated in *Sub1* KO BMDMs (Figure 5E). Consistent with the increased cholesterol efflux activity of *Sub1* KO BMDMs, *Sub1* KO BMDMs displayed upregulation of the cholesterol transporters ATP‐Binding Cassette Sub‐Family G Member 1 (Abcg1) and ATP‐Binding Cassette Subfamily A Member 1 (Abca1) coupled with downregulation of oxLDL Receptor 1 (Olr1) (Figure 5F).

Increases in free cholesterol accumulation within macrophages lead to an inflammatory phenotype and an increased likelihood of foam cell formation.^[^ ^19^ ^]^ Under acetylated LDL (acLDL)‐treated conditions, accumulation of total cholesterol (TC), cholesterol ester (CE), and free cholesterol (FC) all decreased in *Sub1* KO BMDMs (Figure 5G). Nevertheless, the ratio of esterified/free cholesterol (CE/FC) did not significantly change in *Sub1* KO BMDMs, indicating no significant effect on cholesterol mobilization and storage (Figure 5H). The above results indicate that *Sub1* KO lowers macrophage cholesterol accumulation under acLDL‐treated conditions.

Mice on high‐fat diets (HFDs) typically show elevated serum LDL‐C levels due to the inflammatory response of liver macrophages;^[^ ^20^ ^]^ therefore, we hypothesized that myeloid *Sub1* KO in HFD‐fed mice would lower serum LDL‐C levels. Indeed, HFD‐fed *Sub1* KO mice showed lower serum LDL‐C levels relative to their WT and HEMI counterparts (Figure S6, Supporting Information). The above results indicate that *Sub1* KO in macrophages lowers circulating LDL‐C levels under HFD conditions.

### 2.6 Transplantation of *Sub1* Knockout Macrophages Reduces Western Diet‐Induced Atherosclerosis in *Ldlr* ^−/−^ Mice

The consumption of a Western diet (i.e., high cholesterol and saturated fatty acid content) contributes to enhanced circulating cholesterol levels, oxLDL‐induced macrophage activation, and pathogenic foam cell differentiation.^[^ ^21^ ^]^ To analyze the role of *Sub1*‐deficient macrophages in Western diet‐induced atherosclerosis in vivo, we examined the effect of transplanting transgenic bone marrow into a Western diet‐fed *Ldlr* ^−/−^ murine model of atherosclerosis. Irradiated *Ldlr* ^−/−^ mice transplanted with either *Sub1* ^flox/flox^ bone marrow (WT→*Ldlr* ^−/−^), *LysM* ^Cre/−^/*Sub1* ^flox/wt^ bone marrow (HEMI→*Ldlr* ^−/−^), *LysM* ^Cre/−^/*Sub1* ^flox/flox^ bone marrow (*Sub1* KO→*Ldlr* ^−/−^), or *LysM* ^Cre/−^/*Sub1* ^flox/flox^; Signal Transducer And Activator Of Transcription 6 (*Stat6*)^−/−^ bone marrow (*Sub1*, *Stat6* KO→*Ldlr* ^−/−^) were fed a Western diet for 12 weeks. As Stat6 signaling is a key mediator of M2 macrophage polarization,^[^ ^22^ ^]^ we used *Sub1*, *Stat6* KO→*Ldlr* ^−/−^ mice to assess whether *Sub1* KO's effects were primarily mediated through M2 polarization. Immunoblotting confirmed Sub1 and Stat6 knockdown in the appropriate bone marrow samples prior to transplantation (Figure S7, Supporting Information). There were no significant differences in body weight or lipid profiles in the four cohorts (Figure S8A–E, Supporting Information), probably due to the effects of *Ldlr* ^−/−^ phenotype in increasing LDL‐C levels.

**Figure 6:**
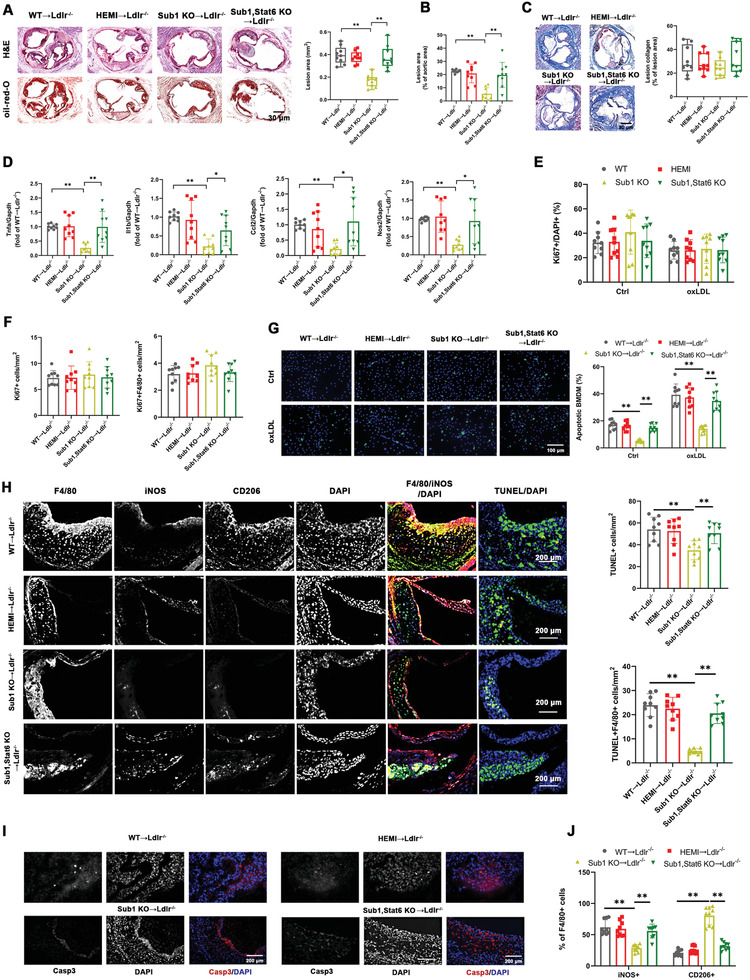
Transplantation of *Sub1* knockout macrophages reduces Western diet‐induced atherosclerosis in *Ldlr* ^−/−^ recipient mice. Irradiated *Ldlr* ^−/−^ mice transplanted with bone marrow from *Sub1* ^flox/flox^ mice (WT→*Ldlr* ^−/−^), *LysM* ^Cre/−^/*Sub1* ^flox/wt^ mice (HEMI→*Ldlr* ^−/−^), *LysM* ^Cre/−^/*Sub1* ^flox/flox^ mice (*Sub1* KO→*Ldlr* ^−/−^), or *LysM* ^Cre/−^/*Sub1* ^flox/flox^; *Stat6* ^−/−^ mice (*Sub1*, *Stat6* KO→*Ldlr* ^−/−^) were fed a Western diet for 12 weeks. A) Representative H&E and Oil Red O staining images showing lesion areas in aortic root sections. Scale bar = 200 µm. Quantification of aortic root lesion areas based on 8–12 10 µm sections per mouse (30 µm apart). B) Quantification of total lesion areas in en face aortas. C) Representative Masson's trichrome staining images and quantification of aortic root lesion collagen by Zeiss Axiovision software. Scale bar = 200 µm. D) qPCR of M1 marker genes in isolated aortic root plaque macrophages. E) In vitro bone marrow‐derived macrophages (BMDMs) proliferation under vehicle (Ctrl) or oxLDL (50 µg mL^−1^, 24 h) conditions and F) in vivo F4/80+ macrophage proliferation in aortic root lesions examined using anti‐Ki67 immunofluorescence. Scale bar = 100 µm. G) In vitro BMDM apoptosis levels under vehicle (Ctrl) or oxLDL (50 µg mL^−1^, 24 h) conditions assessed by TUNEL staining. Cell morphology analyzed by differential interference contrast (DIC) and nuclear staining by DAPI. Apoptotic cell percentage expressed as ratio of TUNEL+/DAPI+. Scale bar = 100 µm. *n* = 9 fields per group. H,I) In vivo F4/80+ macrophage apoptosis in aortic root lesions examined by TUNEL and cleaved caspase‐3 staining. Scale bar = 100 µm. J) Immunofluorescent staining analysis of M1 macrophages (iNOS+/F4/80+) and M2 macrophages (CD206+/F4/80+) in serial aortic root lesion sections. Data reported as means ± SDs. In vivo experiments: *n* = 9 mice per group. In vitro experiments: *n* = 3 biological replicates × 3 technical replicates. *p < 0.05 and **p < 0.01 (A–D,F,H,J: one‐way ANOVA with Fisher's LSD; E,G: two‐way ANOVA with Fisher's LSD; comparing *n* = 3 in vitro biological replicates per group or *n* = 9 mice per group).

*Sub1* KO→*Ldlr* ^−/−^ aortic root atherosclerotic lesions were considerably smaller than the two control cohorts; notably, the addition of *Stat6* KO abrogated the antiatherosclerotic effects of *Sub1* KO (**Figure** **6**A,B). Lesion collagen deposition (as detected by Masson's trichrome stain) was similar in the four cohorts (Figure 6C). Plaque macrophages from *Sub1* KO→*Ldlr* ^−/−^ lesions displayed decreased proinflammatory M1 marker expression, effects abrogated by *Stat6* KO (Figure 6D). By Ki67 immunofluorescence, there were no significant differences in macrophage proliferation in vitro or in atherosclerotic lesions in vivo among the four cohorts (Figure 6E,F). However, in vitro TUNEL assay showed decreased apoptosis levels in *Sub1* KO macrophages under basal conditions and oxLDL challenge, which were abrogated by *Stat6* KO (Figure 6G). Moreover, in vivo TUNEL assay and cleaved caspase‐3 staining detected lower apoptosis levels in *Sub1* KO→*Ldlr* ^−/−^ lesions, effects abrogated in *Sub1*, *Stat6* KO→*Ldlr* ^−/−^ lesions (Figure 6H,I). In addition, *Sub1* KO→*Ldlr* ^−/−^ atherosclerotic lesions displayed decreased proinflammatory iNOS+ M1 macrophage content coupled with increased anti‐inflammatory CD206+ M2 macrophage content, effects abrogated in *Sub1*, *Stat6* KO→*Ldlr* ^−/−^ lesions (Figure 6J). These data indicate that macrophage *Sub1* KO reduces atherosclerosis primarily through promoting anti‐inflammatory M2 polarization as opposed to affecting macrophage proliferation or apoptosis.

As macrophage infiltration into the myocardium can produce ventricular hypertrophy,^[^ ^23^ ^]^ we investigated any possible effects of macrophage *Sub1* KO on the murine myocardium. We noted no significant differences in the interventricular septum or ventricular myocardium among the four cohorts (Figure S9A, Supporting Information). We also observed no signs of aortic valve calcification by Alizarin Red staining among the four cohorts (Figure S9B, Supporting Information). We also found no significant differences in macrophage infiltration within the myocardium among the four cohorts (Figure S9C, Supporting Information).

### 2.7 Macrophage Sub1 Upregulates M1‐Skewing Irf1 Expression in a Ck2‐Dependent Manner

**Figure 7:**
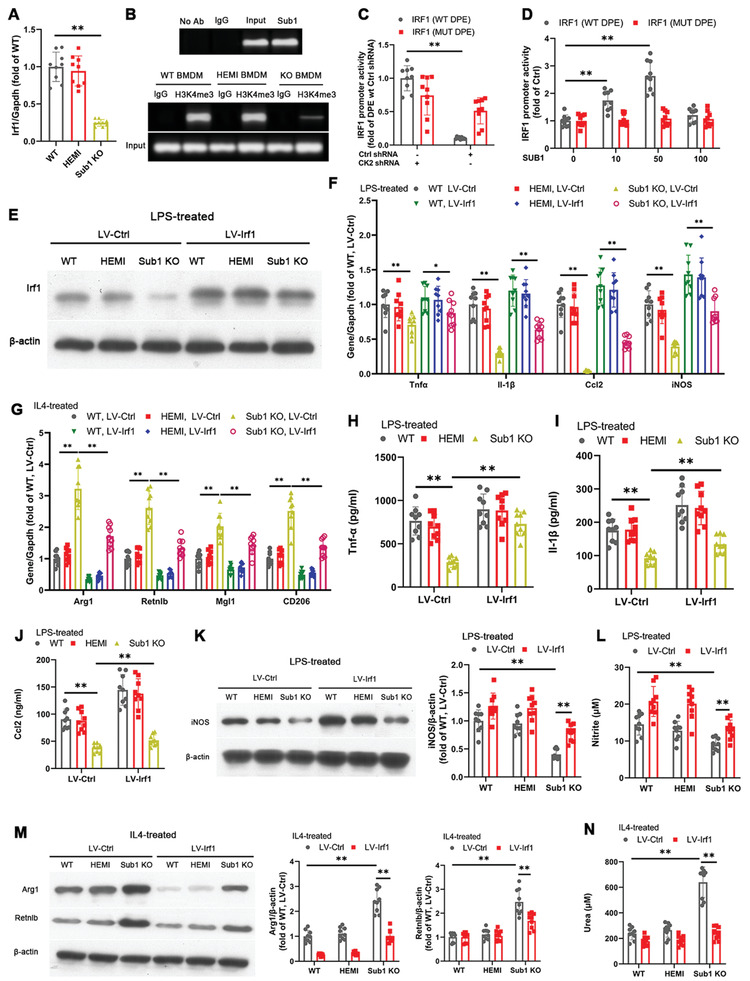
Macrophage Sub1 upregulates M1‐skewing Irf1 expression in a Ck2‐dependent manner. Unless otherwise stated, the following experiments employed *Sub1* ^flox/flox^ (wild‐type, WT), *LysM* ^Cre/−^/*Sub1* ^flox/wt^ (hemizygous, HEMI), and *LysM* ^Cre/−^/*Sub1* ^flox/flox^ (knockout, KO) bone marrow‐derived macrophages (BMDMs). A) qPCR of *Irf1* mRNA levels. B) Chromatin immunoprecipitation (ChIP)‐based detection of Sub1's interaction with the *Irf1* promoter in WT BMDMs (top). Open confirmation of *Irf1* promoter detected by H3K4me3 antibody ChIP in BMDMs (bottom). Human THP‐1 cells were transfected with the WT *IRF1* promoter or mutant (MUT) *IRF1* promoter alone or in combination with C) CK2 shRNA or scrambled control shRNA, or D) Lenti‐GIII‐CMV‐CK2‐HA or control vector. Renilla luciferase vector was cotransfected as normalization control. Luciferase reporter activity was detected 48 h post‐transfection. E–N) BMDMs were transfected with Lenti‐GIII‐CMV‐*Irf1*‐HA (LV‐Irf1) to enable stable Irf1 overexpression. Where indicated, BMDMs were stimulated with M1‐polarizing LPS (100 ng mL^−1^, 8 h) or M2‐polarizing IL4 (5 ng mL^−1^, 8 h). E) Immunoblotting of Irf1. F) qPCR of M1 marker genes under vehicle (Ctrl) or LPS conditions. G) qPCR of M2 marker genes under vehicle (Ctrl) or IL4 conditions. ELISA of H) Tnf‐*α*, I) Il‐1*β*, and J) Ccl2 secretion under vehicle (Ctrl) or LPS conditions. K) Immunoblotting of iNOS protein expression and L) nitrite‐based iNOS activity under vehicle (Ctrl) or LPS conditions. M) Immunoblotting of Arg1 and Retnlb protein expression and N) urea‐based Arg1 activity under vehicle (Ctrl) or IL4 conditions. Data reported as means ± SDs. *n* = 3 biological replicates × 3 technical replicates. *p < 0.05 and **p < 0.01 (A: one‐way ANOVA with Fisher's LSD; B–N) two‐way ANOVA with Fisher's LSD; comparing *n* = 3 in vitro biological replicates per group).

The proinflammatory transcription factor Irf1 is known to play a role in regulating several proteins involved in atherosclerosis‐related inflammation, such as Retinoic Acid‐Inducible Gene I (RIG‐I) and Interleukin 8 (Il‐8).^[^ ^24^ ^]^ Sub1 is phosphorylated by the protein kinase Ck2, which is induced by LPS and is associated with proinflammatory downstream gene expression in macrophages.^[^ ^25^ ^]^ Moreover, the combination of Sub1 and Ck2 is necessary for downstream promoter element (DPE)‐dependent transcription of *Irf1*.^[^ ^26^ ^]^ Therefore, we hypothesized that Irf1 may be a key downstream intermediary for Sub1's effects on atherogenesis. Consistent with our immunoblotting findings (Figures 2D and 5A), we confirmed *Irf1* mRNA downregulation in *Sub1* KO BMDMs by qPCR (**Figure** **7**A). For in vivo validation, we confirmed that C29 or TAK‐242 reduced *Irf1* mRNA expression in macrophages isolated from chow‐fed *ApoE* ^−/−^ WT murine aortic root plaques (Figure S10A, Supporting Information). Conversely, Pam or LPS enhanced *Irf1* mRNA expression in plaque macrophages from chow‐fed *ApoE* ^−/−^ WT and HEMI mice, effects abrogated in *ApoE*, *Sub1* KO mice (Figure S10B, Supporting Information). Moreover, *Irf1* mRNA expression was downregulated in Western diet‐fed *Sub1* KO→*Ldlr* ^−/−^ aortic root plaque macrophages (Figure S10C, Supporting Information). ChIP studies with an anti‐Sub1 antibody confirmed that BMDMs possess Sub1 expression at the *Irf1* promoter (Figure 7B). Moreover, ChIP studies with an anti‐trimethylated lysine 4 of histone 3 (H3K4me3) antibody, a marker of open chromatin structure, suggests reduced *Irf1* promoter activity in *Sub1* KO BMDMs (Figure 7B).

To ascertain whether Sub1's promotion of *Irf1* expression is Ck2‐dependent, we first generated luciferase reporter constructs of human *IRF1* with WT and mutant DPE binding sites. The WT DPE binding site construct showed decreased luciferase reporter activity with shRNA‐induced CK2 knockdown; however, no significant difference was observed with the mutant DPE binding site construct (Figure 7C). CK2 overexpression dose‐dependently increased luciferase reporter activity of the WT DPE binding site construct, but had no effect on the mutant DPE binding site construct (Figure 7D). This evidence indicates that SUB1 promotes *IRF1* expression in a CK2‐dependent manner.

Seeing that Sub1 promotes *Irf1* expression in BMDMs, we examined whether lentiviral Irf1 overexpression (Figure 7E) could rescue the effects of *Sub1* KO on BMDM polarization. Irf1 overexpression in *Sub1* KO BMDMs upregulated proinflammatory M1 marker expression under LPS conditions (Figure 7F) but downregulated anti‐inflammatory M2 marker expression under IL‐4 conditions (Figure 7G). Irf1 overexpression in *Sub1* KO BMDMs also upregulated Tnf‐*α*, Il‐1*β*, and Ccl‐2 secretion under LPS conditions (Figure 7H–J). Moreover, Irf1 overexpression in *Sub1* KO BMDMs enhanced iNOS protein expression and activity under LPS conditions (Figure 7K,L) but decreased Arg1 and Retnlb protein expression as well as Arg1 activity under IL‐4 conditions (Figure 7M,N). Irf1 overexpression reversed cholesterol handling in *Sub1* KO BMDMs (Figure S11A,B, Supporting Information). Irf1 overexpression downregulated Abca1 and Abcg1 expression but upregulated Olr1 levels in cholesterol‐loaded *Sub1* KO BMDMs (Figure S11C, Supporting Information). These results indicate that Sub1‐induced Irf1 activity is an important contributor to the heightened inflammatory response and dysfunctional cholesterol handling in macrophages.

## 3 Discussion

Inflammatory macrophage polarization increases the propensity of atherosclerosis.^[^ ^15^ ^]^ However, the molecular mechanisms underlying this phenomenon are not clearly understood. Herein, we sought to identify master regulator TFs regulating the macrophage TLR transcriptional signature in atherosclerosis. We accomplished this in silico analysis in two stages; first we employed TLR2‐ and TLR4‐stimulated and control macrophages to identity the TLR2 and TLR4 transcriptional signatures in macrophages. Second, we used six publicly available whole transcriptome datasets on atherosclerosis patient‐derived carotid specimens to generate an integrated gene coexpression network. Superimposition of the TLR transcriptional signatures with the coexpression network identified three master regulons common to both TLR signatures: *SUB1*, the NF‐kB subunit *REL*, and *TAF10*. As a novel master regulon in atherosclerosis, we further investigated the role of macrophage Sub1 in atherosclerosis. Our follow‐up experiments revealed that the proatherosclerotic effects of TLR2 and TLR4 signaling in chow‐fed *ApoE* ^−/−^ mice are mediated by Sub1. Moreover, *Sub1* KO macrophages show reduced atherogenic characteristics such as anti‐inflammatory M2 skewing and improved cholesterol handling. In irradiated Western diet‐fed *Ldlr* ^−/−^ mice, macrophage *Sub1* KO reduced atherosclerotic burden. We also showed that expression of the proinflammatory transcription factor Irf1 was reduced upon *Sub1* KO and artificially restoring Irf1 expression in *Sub1* KO macrophages reversed *Sub1* KO's positive effects on macrophage polarization and cholesterol handing. This combined evidence identifies *Sub1* as a master regulon of the atherogenic TLR response in macrophages.

Macrophage accumulation within the arterial wall is a prominent feature in atherosclerotic plaques.^[^ ^2^ ^]^ Exposure to various stimuli within the plaque microenvironment (e.g., TLR ligands, cytokines, oxidized lipids, etc.) can skew the polarization of plaque macrophages toward a proinflammatory M1 phenotype (classically activated by lipopolysaccharide and/or IFN‐ɣ exposure) and away from an anti‐inflammatory M2 phenotype (alternatively activated by IL‐4 or IL‐13 exposure).^[^ ^13^ ^]^ It is well‐recognized that skewing favoring M1 over M2 polarization is a key element in atherosclerotic progression;^[^ ^15^ ^]^ M1 macrophages secrete proinflammatory cytokines and promote atherogenesis and plaque vulnerability while M2 macrophages secrete anti‐inflammatory cytokines and promote atheroprotection.^[^ ^15^ ^]^ Consistent with this inflammatory model of atherogenesis, we observed that TLR2 and TLR4 signaling are proinflammatory and proatherosclerotic in chow‐fed *ApoE* ^−/−^ mice and that these proatherosclerotic effects are mediated by Sub1. Moreover, we found that *Sub1* KO increased M2 marker expression (i.e., CD206, Arg1, Mgl1, and Retnlb)^[^ ^17^ ^]^ and decreased M1 marker expression (i.e., Tnf*α*, Il‐1*β*, Ccl2, and iNOS)^[^ ^17^ ^]^ in macrophages under their respective priming conditions. These findings indicate that *Sub1* supports skewing of the M1/M2 polarization balance toward a proinflammatory, proatherogenic M1 state. Recognizing that *Sub1* is a master regulon of TLR signaling in macrophages, these results are consistent with TLR2 or TLR4 signaling activation also skewing macrophages toward the M1 phenotype.^[^ ^27^ ^]^

In addition to secreting inflammatory mediators, plaque macrophages can absorb oxLDL to form atherogenic foam cells or, contrarily, efflux cholesterol through reverse cholesterol transport to reverse plaque progression.^[^ ^28^ ^]^ Although oxLDL exposure does not skew macrophages toward either M1 or M2,^[^ ^29^ ^]^ macrophage polarization can adversely affect their cholesterol handling. For instance, the M1 macrophage‐secreted cytokine Il‐1*β* lowers ApoE‐mediated cholesterol efflux and cholesterol transporter expression,^[^ ^30^ ^]^ leading to foam cell formation. Here, we observed that *Sub1* KO macrophages showed decreased proinflammatory cytokine secretion coupled with enhanced ApoE‐mediated cholesterol efflux and cholesterol transporter expression under cholesterol‐loading conditions. In vivo, *Sub1* KO→*Ldlr* ^−/−^ atherosclerotic plaques displayed decreased size, enhanced M2 macrophage content, and decreased M1 macrophage content, all of which were abrogated by KO of the M2‐polarization transcription factor Stat6. Therefore, anti‐inflammatory M2 skewing contributes to the observed reduction in atherosclerotic burden in *Sub1* KO→*Ldlr* ^−/−^ mice.^[^ ^3^ ^]^ Validating these findings, macrophage *Sub1* KO on an *ApoE* ^−/−^ background also reduced atherosclerotic burden and promoted M2 skewing. These results support Sub1's role in promoting a proinflammatory, proatherogenic environment via M1 skewing.

The phagocytosis of apoptotic cells by macrophages, a process termed efferocytosis, also plays a key role in controlling plaque inflammation and atherosclerotic progression.^[^ ^31^ ^]^ Efferocytosis reduces the release of danger‐associated molecular pattern molecules (DAMPs) by necrotic cells and also suppresses proinflammatory responses in macrophages.^[^ ^31^ ^]^ Macrophage polarization can also adversely affect the proper efferocytosis within plaques, with M2 macrophages displaying enhanced efferocytosis abilities relative to M1 macrophages.^[^ ^32^ ^]^ Here, we observed lower apoptotic indices in *Sub1* KO→*Ldlr* ^−/−^ plaques, which were abrogated by *Stat6* KO. Thus, these lower plaque apoptosis levels may be attributed to *Sub1* KO‐mediated increases in plaque M2 macrophage content. These results also support *Sub1*’s role in promoting a proinflammatory, proatherogenic environment via M1 skewing.

The family of interferon regulatory factors (IRFs) play a critical role in monocyte lineage development, monocyte‐to‐macrophage differentiation, and M1/M2 polarization.^[^ ^33^ ^]^ Specifically, Irf1 (as well as Irf5 and Irf8) are associated with M1 polarization, while Irf3 and Irf4 are associated with M2 polarization.^[^ ^33^ ^]^ More recent work has revealed that LPS/TLR4‐induced, HDL‐C‐regulated DEGs display a strong enrichment for NF*κ*B p65‐ and Irf1‐dependent genes, and HDL‐C's proinflammatory effects in macrophages require NF*κ*B p65 and are partly dependent on Irf1.^[^ ^34^ ^]^ Consistent with this evidence, our in silico analysis found that the NF‐kB subunit *REL* and the upstream effector of IRF1—*SUB1*—are master regulons of TLR signaling in macrophages and that SUB1, as an upstream effector of IRF1, is associated with M1 polarization. As we show that Irf1 overexpression is able to rescue the effects of *Sub1* KO in macrophages, our study is the first to suggest that the Sub1/Irf1 axis may be a critically active pathway in proatherogenic, TLR‐induced M1 polarization within plaque macrophages.

There are a number of limitations to our findings. First, atherosclerotic plaques derived from different vascular compartments or those of varying stages or stability can significantly differ in terms of macrophage polarization.^[^ ^35^ ^]^ Therefore, the evidence from heterogeneous carotid specimens presented here cannot be generalized to all atherosclerotic plaques. Second, the data accompanying the GEO datasets employed in our bioinformatics analysis did not include important factors such as treatment history, patient survival, and mutational burden. Third, for the carotid specimens whose transcriptomic profiles were included in our bioinformatics analysis, we could not obtain the associated tissue samples for histological analysis to verify the conclusions drawn by our bench studies. Fourth, we did not fully analyze the effects of Irf1 in the context of *Sub1* KO using in vivo models. Future research should pursue this line of enquiry.

## 4 Conclusion

In conclusion, this study demonstrates how in silico identification of disease‐associated master regulons can be a useful method to pinpoint key master TFs. We also demonstrate the role of the TLR master regulon Sub1, and its downstream effect on the transcription factor Irf1, in promoting proinflammatory M1 polarization and atherosclerosis. Our findings suggest that targeting the SUB1/IRF1 axis may be an effective strategy toward combating proatherogenic, TLR‐induced M1 polarization.

## 5 Experimental Section

### Experimental Design

The transcriptional master regulator technique, a method combining transcription signatures with coexpression analysis,^[^ ^36^ ^]^ was applied to pinpoint master regulator TFs. The goals were accomplished in three stages, which are outlined below: i) extraction of TLR transcription signatures for TLR2 and TLR4 from whole transcriptome data, ii) determination of master regulator TFs from the two TLR signatures, and iii) follow‐up in vitro and in vivo studies on the novel master regulon *SUB1*. Please refer to the Supporting Information for the scripts and coding employed in steps (i) and (ii).

### Extraction of TLR Transcription Signatures from Whole Transcriptome Data

Pam is a bacterial lipopeptide mimic that stimulates TLR2 signaling, while Gram‐negative bacterial LPS stimulates TLR4 signaling.^[^ ^37^ ^]^ Microarray data from whole transcriptome analyses of i) Pam‐treated versus nontreated mouse BMDMs cultures at 8 h of exposure (for the TLR2 analysis) and ii) LPS‐treated versus nontreated BMDMs cultures at 8 h of exposure (for the TLR4 analysis) were extracted from the Gene Expression Omnibus (GEO) database (accession number: GSE89988). This microarray data was derived using an Illumina TotalPrep RNA Amplification Kit (Ambion, Grand Island, NY) and a MouseWG‐6 v2.0 R2 Expression BeadChip (Illumina, San Diego, CA) that contains 45 281 probes. The 8 h timepoint was chosen as steady‐state transcript levels peak around the 6 h mark during the murine macrophage response to TLR stimuli.^[^ ^38^ ^]^ The Bioconductor Limma package in R was used to identify DEGs in i) Pam‐treated versus nontreated BMDMs and ii) LPS‐treated versus nontreated BMDMs. BioMart (Ensembl) was used to map murine Ensembl IDs to human Ensembl IDs.

### Determination of Master Regulator TFs from Integrating the TLR Transcription Signatures and Atherosclerosis Datasets

Herein, the transcriptional master regulator technique was applied, which first required the construction of a coexpression network in atherosclerosis, followed by identification of the master regulator TFs that generate the TLR2 and TLR4 signatures. First, six independent transcriptome datasets comprising 371 patient‐derived carotid specimens were retrieved from the GEO database: GSE43292 (*n* = 32 plaques and 32 normal tissue; GPL6244 Human Gene 1.0 ST Array platform, Affymetrix, Santa Clara, CA), GSE24495 (*n* = 113 plaques; GPL10687 Rosetta/Merck Human RSTA 1.0 Array platform, Affymetrix), GSE21545 (*n* = 126 plaques; GPL570 Human Genome U133 Plus 2.0 Array platform, Affymetrix), GSE28829 (*n* = 16 advanced plaques; GPL570 Human Genome U133 Plus 2.0 Array platform, Affymetrix), GSE13922 (*n* = 11 plaques; GPL6255 humanRef‐8 v2.0 Expression BeadChip platform, Illumina), and GSE100927 (*n* = 29 plaques and 12 normal tissue; GPL17077 Agilent‐039494 SurePrint G3 Human GE v2 8x60K Microarray platform, Agilent Technologies, Palo Alto, CA). The whole transcriptome data were preprocessed prior to analysis. First, Affy and affyPLM (Bioconductor) were used to perform quality control (QC) on the Affymetrix datasets.^[^ ^39^ ^]^ Briefly, affyPLM fits the probe‐level robust regressions (probe‐level model, PLM) to produce probe‐set summaries. The relative log expression (RLE) and normalized unscaled standard error (NUSE) values were derived from the PLM in order to quantitatively assess array quality. RLE values were computed for each probe‐set in the array by calculating the ratio of the expression of a particular probe‐set and the median expression of each probe‐set across all arrays. Since most probes were not expected to change across arrays, the distribution of the log‐ratios should be centered around zero. The NUSE values represent the individual probe standard error (SE) from the PLM fit. The SE values were normalized at the probe‐level across arrays so that the distribution of SE values across arrays should be centered around unity. Then, robust multiarray averaging (RMA) was used to normalize microarray data from the Affymetrix datasets.^[^ ^40^ ^]^ Due to the presence of multiple platforms, ComBat was then used to eliminate batch effects within the samples.^[^ ^41^ ^]^

Using the processed data, a coexpression analysis followed by master regulon identification was performed for the TLR2 and TLR4 gene networks. Briefly, the Bioconductor RTN package in R was employed for transcriptional network inference and analysis.^[^ ^36^ ^]^ Coexpression analysis was executed with the corpcor partial correlation package (CRAN R project) using the pcor.shrink tool in default settings.^[^ ^9^ ^]^ The fdrtool package (CRAN R project) was utilized to estimate the false discovery rate (FDR) and statistical significance of every partial correlation.^[^ ^36^ ^]^ VIPER analysis based on multiple‐sample gene expression signatures (the Bioconductor msVIPER module) was used to identify the master regulons in each gene network.^[^ ^42^ ^]^ The igraph fastgreedy.community module (CRAN R project) was used to determine community structure for the TLR2 and TLR4 gene networks.^[^ ^43^ ^]^

### Gene Set Enrichment Analysis for the Gene Communities

GSEA was performed on the gene communities identified within the TLR2 and TLR4 gene networks. The GSEA was performed against multiple ontologies: GO terms (including molecular function (MF), cellular component (CC), and biological process (BP)), Kyoto Encyclopedia of Genes and Genomes (KEGG), Reactome (REAC), Transfac (TF), miRTarBase (MIRNA), CORUM protein complexes (CORUM), Human Phenotype Ontology (HP), Human Protein Atlas (HPA), and Online Mendelian Inheritance in Man (OMIM).

### Animal Models

All animal experimental procedures were approved by the Institutional Animal Care and Use Committee of the Second Affiliated Hospital of Chongqing Medical University (no. 2019653). Wild‐type C57BL/6, *Ldlr* ^−/−^, *ApoE* ^−/−^, and *Stat6* ^−/−^ mice on a C57BL6 genetic background were purchased from the Jackson Laboratory (Bar Harbor, ME). First, mice possessing a floxed allele of *Sub1* (*Sub1* ^flox/flox^; herein termed WT) were created and then these were crossed with *LysM* ^Cre^ mice expressing Cre‐recombinase in the myeloid lineage to produce *LysM* ^Cre/−^/*Sub1* ^flox/flox^ (herein termed *Sub1* KO) mice and myeloid‐specific hemizygous *LysM* ^Cre/−^/*Sub1* ^flox/wt^ (herein termed HEMI) mice.^[^ ^44^ ^]^ Then, *Sub1*, *Stat6* KO mice and *ApoE*, *Sub1* KO mice were created by crossbreeding *Sub1* KO mice with *Stat6* ^−/−^ or *ApoE* ^−/−^ mice, respectively. Myeloid lineage‐specific knockout and germline transmission were confirmed by PCR using the following genotyping primers: *Lox* gt forward, 5′‐GTG CTC CTT AAG TGT TGC AG‐3′; *Lox* gt reverse, 5′‐CCC TTC ATG TAA GTA TTC TC‐3′; *Frt* gt forward, 5′‐GAC TCT TGG ACA GCC AAG CTC‐3′; and *Frt* gt reverse, 5′‐TTT CAT GAT GCC TGG CCT TTC.

Unless otherwise specified, mice were provided standard rodent chow (44.2% carbohydrates, 6.2% fat, and 18.6% crude protein; T.2018, Harlan, Indianapolis, IN) and tap water ad libitum. The various experimental regimens were initiated at 8 weeks of age. For the TLR inhibitor and agonist studies, male *ApoE* ^−/−^ mice were intraperitoneally (i.p.) administered vehicle (20% DMSO in phosphate‐buffered saline (PBS), Ctrl), C29 (50 mg kg^−1^ daily) (BioVision, Milpitas, CA),^[^ ^45^ ^]^ TAK‐242 (3 mg kg^−1^ daily) (Sigma, St. Louis, MO),^[^ ^46^ ^]^ Pam (15 µg (0.01 µmol) weekly) (Sigma),^[^ ^47^ ^]^ or LPS (50 µg (0.01 µmol) weekly) (Sigma) for 14 weeks.^[^ ^48^ ^]^ For lipid metabolic studies, male WT, HEMI, and *Sub1* KO mice were fed standard rodent chow or a HFD (60% fat, TD06414, Harlan) for 20 weeks.

For the bone marrow transplantation model, only male *Ldlr* ^−/−^ donor and male *Ldlr* ^−/−^ recipients were used. Five to seven days before bone marrow transplantation, donor mice and *Ldlr* ^−/−^ recipient mice were provided with antibiotic‐supplemented water. On the day of donor cell injection, recipient mice were exposed to two equal doses of radiation (4.5 Gy for 1.4 min per dose) separated by a 3 h interval. The *Ldlr* ^−/−^ recipient mice received approximately six million bone marrow cells in the retro‐orbital sinus. Animals were fed on regular chow for six weeks after transplantation and, thereafter, with a standardized Western diet (21% kcal from fat and 0.2% cholesterol, TD88137, Harlan) for 12 weeks. To confirm engraftment, recipient animals were anesthetized and perfused with 4% PBS in formaldehyde after drawing blood. The blood was subjected to genotyping for *Cre* transgene.

### Serum Lipid Profiling

Serum levels of total cholesterol, LDL‐C, HDL‐C, and triglycerides were assayed with a test kit (01218LH, Beijing Leadman Biochemistry, Beijing, China) using a Siemens AD‐VIA‐2400 biochemical analyzer (Erlangen, Germany).

### Analysis of Murine Aortic Atherosclerosis

Whole *ApoE* ^−/−^ mice aortas were analyzed by en face Oil Red O staining. For M1 marker analysis, CD14+ macrophages were isolated from aortic root plaques using anti‐CD14 magnetic beads (Invitrogen, Carlsbad, CA) as previously described^[^ ^49^ ^]^ and subjected to qPCR analysis as described below. For aortic root lesion staining, frozen embedded sections of the aortic root starting just distal to the three cusp‐region of the aortic valve were collected from *ApoE* ^−/−^ mice and *Ldlr* ^−/−^ recipient mice. About 70 such sections were acquired, and every third section was subjected to immunohistochemical analysis. 10 µm thick lesion sections (30 µm apart) were stained using hematoxylin and eosin (H & E; PerkinElmer, Waltham, MA), Oil Red O (Sigma), and Masson's Trichome (Sigma), and captured using a RVL‐100 microscope (Echo Laboratories, San Diego, CA). Immunofluorescent staining was performed as described below.

### Immunofluorescent Staining

For cultured BMDM staining, the primary antibodies were as follows: Ki67 (NB500‐170, Novus Biologicals, Littleton, CO) and Galectin‐3 (Mac2) (ab76245, Abcam, Cambridge, MA). For staining of aortic root sections, the following primary antibodies were used: EGF‐like module‐containing mucin‐like hormone receptor‐like 1 (F4/80) (PA5‐32399, Invitrogen), iNOS (ab3523, Abcam), CD206 (ab64693, Abcam), cleaved caspase‐3 (9661, Cell Signaling Technology (CST), Danvers, MA), CD68 (ab125212, Abcam), *α*‐actinin (ab137346, Abcam), CD3 (ab16669, Abcam), and Irf1 (8478, CST). The goat antirabbit secondary antibodies Alexa Fluor 647 (red; ab150115, Abcam) and Alexa Fluor 488 (green; ab150077, Abcam) were employed for immunofluorescent staining. A TUNEL Assay Kit (G3250, Promega, Madison, WI) was used to evaluate apoptosis levels in cultured BMDMs and aortic root sections. Cultured BMDMs and aortic root sections were also subjected to 4′,6‐diamidino‐2‐phenylindole (DAPI, Sigma) staining to identify nuclei. Images were captured using a RVL‐100 microscope.

### Murine BMDM Culture

Murine BMDMs were differentiated from bone marrow cells through the use of L929‐conditioned medium. L929‐conditioned medium causes the bone marrow monocyte/macrophage progenitor cells to proliferate and differentiate into mature BMDMs.^[^ ^50^ ^]^ Briefly, the L929‐conditioned medium was prepared as follows: L929 murine fibroblasts (ATCC, Manassas, VA) were collected after seven days of culture, centrifuged at 2000 × *g* for 5 min, and the supernatant was filtered using a 0.45 µm low protein binding filter. Bone marrow cells from murine femurs and tibias were obtained by flushing the marrow tissue with Dulbecco's Modified Eagle's Medium (DMEM; GE Hyclone, Pittsburgh, PA), centrifuging at 1400 × *g* for 5 min, and resuspension. Bone marrow cells were differentiated into mature BMDMs in DMEM supplemented with 20% fetal bovine serum (FBS), 15% L929‐conditioned medium, penicillin/streptomycin, and l‐glutamine on nontissue culture treated plates (BD Falcon, Franklin Lakes, NJ) for six days, with medium changed at day in vitro (DIV) 4.

Mature BMDMs were exposed to PBS vehicle control, Pam (100 ng mL^−1^), LPS (100 ng mL^−1^), or IL‐4 (5 ng mL^−1^, Sigma) for 8 h prior to analysis. Enzyme‐linked immunosorbent assay (ELISA) was used to measure Tnf‐*α*, Il‐1*β*, and Ccl‐2 secreted into the media. For modified LDL experiments, the BMDMs were exposed to 30 µg mL^−1^ oxLDL (Yiyuan Biotechnology, Guangzhou, China) or 50 µg mL^−1^ acLDL (Yiyuan Biotechnology) for 24 h prior to analysis.

### Cholesterol Accumulation, Uptake, Efflux, and Content Assays

BMDMs were oxLDL‐exposed, stained, and visualized for cholesterol accumulation. Briefly, coverslip‐grown BMDMs were treated with oxLDL (30 µg mL^−1^) for 24 h, fixed in 4% paraformaldehyde (in PBS) after washing, washed with PBS followed by 60% isopropanol and incubated with 0.3% Oil Red O (in 60% isopropanol) for 1 h, and washed two times in PBS and again once with 60% isopropanol. DAPI was used to stain the BMDMs. Images were captured using a RVL‐100 microscope.

The BMDM cholesterol uptake assay was performed as described previously.^[^ ^51^ ^]^ Briefly, acLDL was preincubated in 1 µCi mL^−1^ [^3^H]‐cholesterol (PerkinElmer) for 16 h at 37 °C. BMDMs were then treated with 50 µg mL^−1^ [^3^H]‐acLDL for 30 min. Cells were washed and treated with room‐temperature 0.5 N NaOH to lyse cells. From each condition, radioactivity aliquots were measured and standardized to BMDM protein obtained from simultaneous culture in different dishes.

The BMDM cholesterol efflux assay was performed as described elsewhere.^[^ ^51^ ^]^ For efflux, BMDMs were treated with 1 µCi mL^−1^ [^3^H]‐cholesterol (PerkinElmer) for 48 h, PBS‐washed, and incubated with serum‐free media (Ctrl) or media containing HDL‐C (50 µg mL^−1^, Yiyuan Biotechnology) or ApoA1 (30 µg mL^−1^, Yiyuan Biotechnology). Four hours later, radioactivity aliquots (150 µL) were measured and standardized‐to‐total cell lysates.

BMDM cholesterol content was measured using the Cholesterol/Cholesteryl Ester Quantification Colorimetric Kit (K603‐100, Biovision, Mountain View, CA) after treating with acLDL (50 µg mL^−1^) for 24 h. CE was calculated by subtracting FC from TC.

### qPCR Analysis

RNA isolation from BMDMs was performed using the RNeasy Mini Kit (74104, Qiagen, Hilden, Germany). An Agilent Bioanalyzer was used to check RNA concentration, integrity, and purity. Routine qPCR was performed using *Gapdh* as an endogenous housekeeping control. Primers used for qPCR are provided in Table S1 in the Supporting Information.

### Immunoblotting

Routine procedures were followed for protein extraction and immunoblotting using *β*‐actin as a loading control.^[^ ^52^ ^]^ The primary antibodies were as follows: *β*‐actin (ab8227, Abcam), p‐Sub1/Sub1 (G‐20, Santa Cruz Biotechnology), iNOS (ab3523, Abcam), Arg1 (ab124917, Abcam), Retnlb (ab11429, Abcam), Abca1 (NB400‐105, Novus Biologicals), Abcg1 (NB400‐132, Novus Biologicals), Olr1 (ab60178, Abcam), and Irf1 (8478, CST). To quantify the band intensities, the films were scanned using a Scanmaker 1000XL (Microtek Lab, Santa Fe Springs, CA). The resulting images were analyzed using an Image Pro Plus v6.1 Analyzer (Media Cybernetics, Rockville, MD).

### iNOS and Arg1 Enzymatic Activity Assays

For assaying iNOS activity, BMDM supernatants were mixed at a 1:1 ratio with Griess reagent (Sigma). Absorbance levels were then measured at 543 nm with a SpectraMax 190 (Molecular Devices, Sunnyvale, CA). The nitrite concentration was calculated with a sodium nitrite 22 standard. For assaying Arg1 activity in BMDMs, the Quantichrome Urea Assay Kit (Bioassay Systems, Hayward, CA) was used.

### Chromatin Immunoprecipitation (ChIP)

ChIP was carried out using standard protocols as previously described.^[^ ^53^ ^]^ The following primary antibodies were used: IgG (ab171870, Abcam), Irf1 (8478, CST), Sub1 (NB100‐59775, Novus Biologicals), and H3K4me3 (ab8580, Abcam).

### Vector Constructs

Human *CK2* shRNA and scrambled control shRNA in pGIPz lentiviral plasmids were obtained from OpenBiosystems (Thermo Scientific, Huntsville, AL). Control lentiviral vector and lentiviral vectors expressing human *CK2* (Lenti‐GIII‐CMV‐*CK2*‐HA) and murine *Irf1* (Lenti‐GIII‐CMV‐*Irf1*‐HA) were obtained from Applied Biological Materials (Richmond, BC, Canada). Human *IRF1* promoter fragments (−1312 to +50) containing either the WT DPE site that enables SUB1 binding (AGACGTG) or a mutant (MUT) DPE site that does not enable SUB1 binding (CTCATGT) were constructed as previously described^[^ ^26^ ^]^ and cloned into an IDTSmart vector (Integrated DNA Technologies, Coralville, IA). The WT and MUT fragments were then subcloned into a pGL3‐basic luciferase reporter plasmid (Promega) to form pGL3‐basic/WT *IRF1* promoter or pGL3‐basic/MUT *IRF1* promoter constructs, respectively. Insert orientations were determined by restriction enzyme digestion and constructs were fully sequenced. Lipofectamine 2000 (Invitrogen) was employed to transfect plasmids according to the kit's instructions.

### Luciferase Reporter Assays

Immortalized human THP‐1 cells were transfected with pGL3‐basic/WT *IRF1* promoter or pGL3‐basic/mutant *IRF1* promoter (750 ng) alone or in combination with *CK2* shRNA or scrambled control shRNA using Lipofectamine 2000. In the second experiment, THP‐1 cells were transfected with pGL3‐basic/WT *IRF1* promoter or pGL3‐basic/mutant *IRF1* promoter (750 ng) alone or in combination with Lenti‐GIII‐CMV‐*CK2*‐HA or control vector. For both experiments, Renilla luciferase control vector (20 ng pRL‐TK, Promega) was cotransfected as control. Luciferase reporter activity was detected 48 h post‐transfection with the Dual Luciferase Reporter Assay System (Promega) via normalization to Renilla luciferase activity.

### Statistical Analysis

Data were presented as means ± standard deviations (SDs) unless stated otherwise. All in vitro experiments consisted of *n* = 3 biological replicates × 3 technical replicates per experimental group and were represented as 9 individual data points. All in vivo studies consisted of *n* = 9 mice per cohort. Statistical tests were performed on data from independent biological replicates (*n* = 3 biological replicates per in vitro experimental group; *n* = 9 mice per in vivo cohort). No data were excluded from the statistical analysis. Normal distributions were validated with the Kolmogorov–Smirnov test. For comparisons between two groups, a two‐tailed unpaired Student's *t*‐test was applied. For comparisons among three or more groups, one‐way ANOVA or two‐way ANOVA followed by Fisher's least significant difference (LSD) post‐hoc testing were applied as indicated. A *p*‐value of less than 0.05 deemed a significance threshold for all analyses. Analyses were performed using SAS Enterprise Guide (v.4.3; SAS Institute) and R (v.3.0.2; R Foundation for Statistical Computing, Vienna, Austria).

## Conflict of Interest

The authors declare no conflict of interest.

## Author Contributions

Y.Q.C., J.L.D., and R.Z.H. conceived and designed the study. R.Z.H., J.L.D., Z.C.H., X.R.C., Y.C., H.R.L., H.Z., Y.Y.L., L.W.L., Y.X.F., Y.W., W.H.S., Z.R.K., Y.Q.C., and N.D.M. performed the experimental procedures. R.Z.H., Y.C., J.L.D., N.D.M., and H.Z. analyzed the data. R.Z.H. and N.D.M. drafted the manuscript.

## Supporting information

Supporting Information

## Data Availability

Research data are not shared.
