# Supplementary material for: The Transcription Factor SUB1 Is a Master Regulator of the Macrophage TLR Response in Atherosclerosis
Source: Adv Sci (Weinh). 2021 Aug 10;8(19):2004162. doi: 10.1002/advs.202004162 (PMC8498911; doi:10.1002/advs.202004162)
Supplement: Supplementary file 1 — Supporting Information [file ADVS-8-2004162-s001.pdf]

WILEY-VCH

© 2021 Wiley-VCH GmbH

ADVANCED SCIENCE
Open Access

Supporting Information

for *Adv. Sci.*, DOI: 10.1002/advs.202004162

The Transcription Factor *SUB1* is a Master Regulator of Macrophage TLR Response in Atherosclerosis

*Rongzhong Huang PhD, Zicheng Hu PhD, Xiaorui Chen MD, Yu Cao PhD, Hongrong Li MD, Hong Zhang MD, Yongyong Li PhD, Liwen Liang MD, Yuxing Feng MD, Ying Wang PhD, Wenhua Su MD, Zerui Kong MD, N.D. Melgiri MD, Lihong Jiang PhD, Xingsheng Li PhD, Jianlin Du PhD**, and *Yunqing Chen PhD**

Supporting Information

The Transcription Factor *SUB1* is a Master Regulator of Macrophage TLR Response in Atherosclerosis

Rongzhong Huang PhD[1], Jianlin Du PhD[2], Zicheng Hu PhD[3], Xiaorui Chen MD[4], Yu Cao PhD[5], Hongrong Li MD[5], Hong Zhang MD[6], Yongyong Li PhD[1], Liwen Liang MD[6], Yuxing Feng MD[7], Ying Wang PhD[8], Wenhua Su MD[6], Zerui Kong MD[9,10], N.D. Melgiri MD[11], Lihong Jiang PhD[5], Xingsheng Li PhD[1], Yunqing Chen PhD[2*]*

SUPPORTING FILES

Software S1. All R codes are provided in a compressed ZIP file located at the following URL: https://drive.google.com/file/d/1TjB82_bbUTOMcYC-wGk4EpGy094DUfUO/view?usp=sharing. This ZIP file contains all R code necessary to replicate the study's analysis as described in the Methods: (i) extraction of TLR transcription signatures for TLR2 and TLR4 from whole transcriptome data, and (ii) determination of master regulator TFs from the two TLR signatures.

File S1. Gene expression profiles of Pam- and LPS-treated versus non-treated BMDMs. Microarray data for Pam-treated (TLR2) and LPS-treated (TLR4) versus non-treated BMDMs.

File S2. Summary statistics for the six co-expression networks. Six co-expression networks were generated from the six transcriptomic datasets derived from human carotid plaques and normal carotid tissue samples (n=371).

File S3. Gene set enrichment analysis (GSEA) of the gene communities within TLR networks. GSEA was applied to the fastgreedy.community-identified gene communities within the TLR2 gene network (n=7 communities) and TLR4 gene network (n=7 communities). Gene Ontology (GO) annotation for Molecular Function (MF) and Biological Processes (BP) and KEGG pathway for significantly enriched terms and pathways by both GSEA and hypergeometric analysis by HTSanalyzeR (www.bioconductor.org/packages/release/bioc/html/HTSanalyzeR.html).

SUPPORTING TABLES

Table S1. qPCR or ChIP analysis primer sequences.

Gene name	Forward primer	Reverse primer
Arg1	GGGAAAGCCAATGAAGAGCTG	AGAAAGGACACAGGTTGC
Ccl2	CACTCACCTGCTGCTACTCATTC	TCTTTGGGACACCTGCTG
CD206	CCCAAGGGCTCTTCTAAAGCA	CGGCACCTATCACA
Gapdh	TCTCCTGCGACTTCAACAGC	TCCAGGGTTTCTTACTTC
iNOS	AGCCCTCACCTACTTCCTG	TCTCTGCCTATCCGTCTC
Il-1β	GCTTCAGGCAGGCAGTATC	ATGGGCTCTTCTTCAAAG
Irf1	TTGCGCCACTTCTCTTAAC	TCCAATCCAGTCTATGTCCC
Mgl1	TGAGAAAGGCTTTAAGAACTGGGGA	CACCTGTAGTGATGTGGG
Retnlb	TCCAGCTAACTATCCCTCCACTGGTG	CCATCTGTTCATAGTCTTGA
Sub1	TTCCAGAGAAGCCCGTGAAG	AAGTCCCGAACACTGACATATC
Tnfα	GGTTCTGTCCCTTTCACTCAC	CTCTTCTGCCAGTTCC
Cre	CCCAGAAATGCCAGATTACG	CTTGGGCTGCCAGAATTTCTC
IRF1 promoter (human)	GGGACAAGGCGGAGTGAGAGG	AGCGGCGAAGGGGAAGTACAG

SUPPORTING FIGURES

Figure S1. Transcriptomic dataset quality control analysis. Quality control (QC) analysis of patient-derived carotid specimen transcriptomic datasets using normalized unscaled standard errors (NUSE) and relative log expression (RLE) values.

Figure S2. Transcriptomic dataset normalization. Pre- and post-robust multi-array averaging (RMA) normalization of microarray probe intensity values from patient-derived carotid specimen transcriptomic datasets.

Figure S3. Schematic of transgenic mouse model construction. Upstream of *Sub1* promoter and downstream of 3'UTR, LoxP sites were inserted in the DNA fragments. For selection of recombinants *Frt* sites flanked by PGK neo cassette was used. Lysozyme promoter driven by Cre recombinase (*LysM*^{Cre}) was used to delete the entire *Sub1* fragment.

Figure S4. Body weights and serum lipid profiling of chow-fed *ApoE*$^{-/-}$ mice cohorts in TLR inhibition experiments. *ApoE*$^{-/-}$; *Sub1*$^{flox/flox}$ (*ApoE*$^{-/-}$ WT) mice were fed a chow diet and administered vehicle (Ctrl), C29 (50 mg/kg), or TAK-242 (3 mg/kg) by daily intraperitoneal (i.p.) injection for 14 weeks. **(A)** Body weights, **(B)** serum total cholesterol, **(C)** serum low-density lipoprotein cholesterol (LDL-C), **(D)** serum high-density lipoprotein cholesterol (HDL-C), and **(E)** serum triglycerides. Data reported as means ± SDs. *n*=9 mice per group. *P<0.05, **P<0.01 [one-way ANOVA with Fisher's LSD].

Figure S5. Body weights and serum lipid profiling of chow-fed *ApoE*$^{-/-}$ mice cohorts in TLR agonism experiments. *ApoE*$^{-/-}$; *Sub1*$^{flox/flox}$ (*ApoE*$^{-/-}$ WT), *ApoE*$^{-/-}$; *LysM*$^{Cre/-}$/*Sub1*$^{flox/wt}$ (*ApoE*$^{-/-}$ HEMI), and *ApoE*$^{-/-}$; *LysM*$^{Cre/-}$/*Sub1*$^{flox/flox}$ (*ApoE*, *Sub1* KO) mice were fed a chow diet and administered vehicle (Ctrl), Pam (15 μg), or LPS (50 μg) by weekly intraperitoneal (i.p.) injection for 14 weeks. **(A)** Body weights, **(B)** serum total cholesterol, **(C)** serum low-density lipoprotein cholesterol (LDL-C), **(D)** serum high-density lipoprotein cholesterol (HDL-C), and **(E)** serum triglycerides. Data reported as means ± SDs. *n*=9 mice per group. *P<0.05, **P<0.01 [two-way ANOVA with Fisher's LSD].

Figure S6. Body weights and serum lipid profiling of HFD-fed mice. *Sub1*$^{flox/flox}$ (wild-type, WT), *LysM*$^{Cre/-}$/*Sub1*$^{flox/wt}$ (hemizygous, HEMI), and *LysM*$^{Cre/-}$/*Sub1*$^{flox/flox}$ (knockout, KO) mice were fed a standard chow or HFD for 20 weeks. **(A)** Body weights, **(B)** serum total cholesterol, **(C)** serum low-density lipoprotein cholesterol (LDL-C), **(D)** serum high-density lipoprotein cholesterol (HDL-C), and **(E)** serum triglycerides. Data reported as means ± SDs. *n*=9 mice per group. *P<0.05, **P<0.01 [two-way ANOVA with Fisher's LSD].

Figure S7. Validation of Sub1 and Stat6 knockdown in *Sub1* KO or *Stat6* KO bone marrow. Immunoblotting of Sub1 and Stat6 in bone marrow samples from *Sub1*$^{flox/flox}$ mice (WT->*Ldlr*$^{-/-}$), *LysM*$^{Cre/-}$/*Sub1*$^{flox/wt}$ mice (HEMI->*Ldlr*$^{-/-}$), *LysM*$^{Cre/-}$/*Sub1*$^{flox/flox}$ mice (*Sub1* KO->*Ldlr*$^{-/-}$), or *LysM*$^{Cre/-}$/*Sub1*$^{flox/flox}$; *Stat6*$^{-/-}$ mice (*Sub1*, *Stat6* KO->*Ldlr*$^{-/-}$) prior to transplantation into irradiated *Ldlr*$^{-/-}$ mice.

Figure S8. Body weights and serum lipid profiling of Western diet-fed *Ldlr*$^{-/-}$ recipient mice. Irradiated *Ldlr*$^{-/-}$ mice transplanted with bone marrow from *Sub1*$^{flox/flox}$ mice (WT->*Ldlr*$^{-/-}$), *LysM*$^{Cre/-}$/*Sub1*$^{flox/wt}$ mice (HEMI->*Ldlr*$^{-/-}$), *LysM*$^{Cre/-}$/*Sub1*$^{flox/flox}$ mice (*Sub1* KO->*Ldlr*$^{-/-}$), or *LysM*$^{Cre/-}$/*Sub1*$^{flox/flox}$; *Stat6*$^{-/-}$ mice (*Sub1, Stat6* KO->*Ldlr*$^{-/-}$) were fed a Western diet for 12 weeks. **(A)** Body weights, **(B)** serum total cholesterol, **(C)** serum low-density lipoprotein cholesterol (LDL-C), **(D)** serum high-density lipoprotein cholesterol (HDL-C), and **(E)** serum triglycerides. Data reported as means ± SDs. *n*=9 mice per group. **P*<0.05, ***P*<0.01 [one-way ANOVA with Fisher's LSD].

Figure S9. Transplantation of *Sub1* KO bone marrow does not significantly impact myocardia of Western diet-fed *Ldlr*$^{-/-}$ recipient mice. *Ldlr*$^{-/-}$ mice transplanted with bone marrow from *Sub1*$^{flox/flox}$ mice (WT->*Ldlr*$^{-/-}$), *LysM*$^{Cre/-}$/*Sub1*$^{flox/wt}$ mice (HEMI->*Ldlr*$^{-/-}$), *LysM*$^{Cre/-}$/*Sub1*$^{flox/flox}$ mice (*Sub1* KO->*Ldlr*$^{-/-}$), or *LysM*$^{Cre/-}$/*Sub1*$^{flox/flox}$; *Stat6*$^{-/-}$ mice (*Sub1, Stat6* KO->*Ldlr*$^{-/-}$) were fed a Western diet for 12 weeks. No discernable evidence of **(A)** hypertrophy in interventricular septa (scale bar=1 mm), **(B)** calcification of aortic valves by Alizarin Red (scale bar=100 μm), or **(C)** macrophage infiltration by CD68+ immunofluorescence (red) on a cardiomyocyte α-actinin+ immunofluorescence (green) background in the four mouse cohorts (scale bar=100 μm). Data reported as means ± SDs. *n*=9 mice per group. **P*<0.05, ***P*<0.01 [one-way ANOVA with Fisher's LSD].

Figure S10. Assessment of *Irf1* mRNA expression in isolated aortic root plaque macrophages. qPCR quantification of *Irf1* mRNA expression in aortic root plaque macrophages isolated from **(A)** *ApoE*$^{-/-}$; *Sub1*$^{flox/flox}$ (*ApoE*$^{-/-}$ WT) mice fed a chow diet and administered (Ctrl), C29 (50 mg/kg), or TAK-242 (3 mg/kg) by daily intraperitoneal (i.p.) injection for 14 weeks, **(B)** *ApoE*$^{-/-}$ WT, *ApoE*$^{-/-}$; *LysM*$^{Cre/-}$/*Sub1*$^{flox/wt}$ mice (*ApoE*$^{-/-}$ HEMI), and *ApoE*$^{-/-}$; *LysM*$^{Cre/-}$/*Sub1*$^{flox/flox}$ mice (*ApoE, Sub1* KO) fed a chow diet and administered vehicle (Ctrl), Pam (15 μg), or LPS (50 μg) by weekly intraperitoneal (i.p.) injection for 14 weeks, and **(C)** *Ldlr*$^{-/-}$ mice transplanted with bone marrow from *Sub1*$^{flox/flox}$ mice (WT-

>$Ldlr^{-/-}$), *LysM*$^{Cre/-}$/*Sub1*$^{flox/wt}$ mice (HEMI->$Ldlr^{-/-}$), *LysM*$^{Cre/-}$/*Sub1*$^{flox/flox}$ mice (*Sub1* KO->$Ldlr^{-/-}$), or *LysM*$^{Cre/-}$/*Sub1*$^{flox/flox}$; *Stat6*$^{-/-}$ mice (*Sub1, Stat6* KO->$Ldlr^{-/-}$) fed a Western diet for 12 weeks. Data reported as means ± SDs. n=9 mice per group. *P<0.05, **P<0.01 [**(A, C)** one-way ANOVA with Fisher's LSD; **(B)** two-way ANOVA with Fisher's LSD].

Figure S11. Improved cholesterol transport in *Sub1* KO macrophages abrogated by Irf1 overexpression. The following experiments employed *Sub1*$^{flox/flox}$ (wild-type, WT), *LysM*$^{Cre/-}$/*Sub1*$^{flox/wt}$ (hemizygous, HEMI), and *LysM*$^{Cre/-}$/*Sub1*$^{flox/flox}$ (knockout, KO) bone marrow-derived macrophages (BMDMs). BMDMs were transfected with Lenti-GIII-CMV-*Irf1*-HA (LV-Irf1) to enable stable *Irf1* overexpression or the matching vector control. **(A)** Cholesterol uptake in BMDMs exposed to [^{3}H] cholesterol-labeled acLDL (50 μg/ml) for 30 min. All values normalized to total protein levels and expressed as fold of WT, LV-Ctrl. **(B)** Cholesterol efflux from BMDMs treated with [^{3}H] cholesterol for 48 h. **(C)** Western blotting of Abca1, Abcg1, and Olr1 in BMDMs following exposure to vehicle (Ctrl) or acLDL (50 μg/ml, 24 h). Data reported as means ± SDs. n=3 biological replicates × 3 technical replicates. *P<0.05, **P<0.01 [two-way ANOVA with Fisher's LSD; comparing n=3 *in vitro* biological replicates per group].

Figure S1

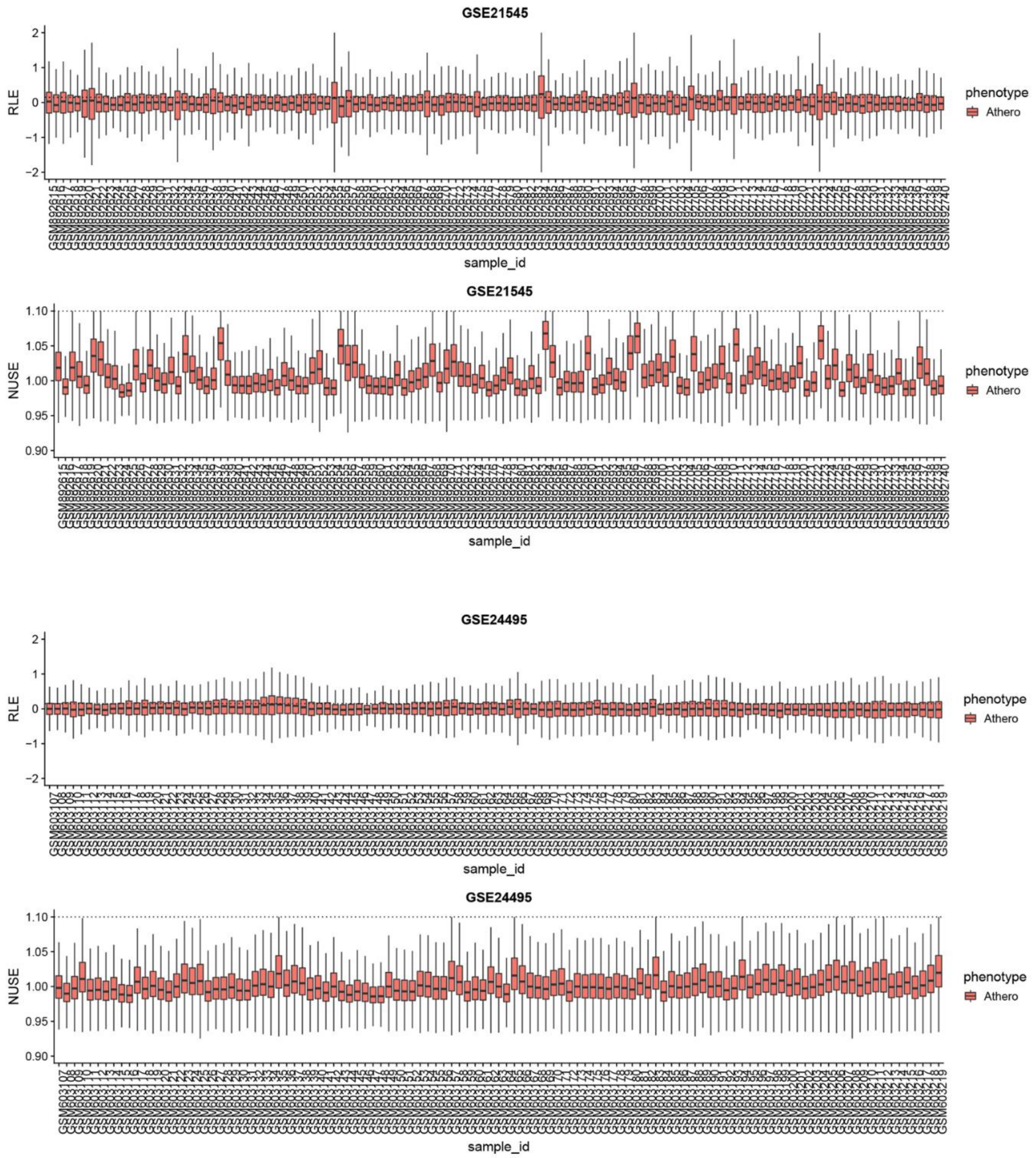
GSE21545
RLE
sample_id
phenotype
Athero
GSE21545
NUSE
sample_id
phenotype
Athero
GSE24495
RLE
sample_id
phenotype
Athero
GSE24495
NUSE
sample_id
phenotype
Athero

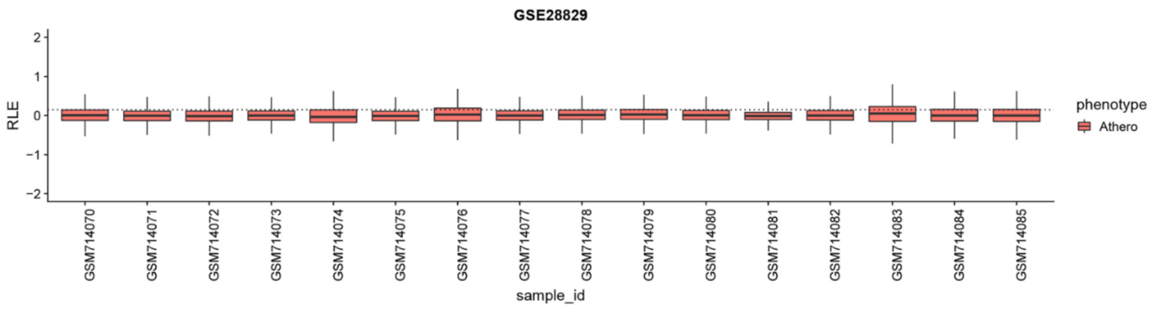
GSE28829
RLE
sample_id
phenotype
Athero
GSM714070 GSM714071 GSM714072 GSM714073 GSM714074 GSM714075 GSM714076 GSM714077 GSM714078 GSM714079 GSM714080 GSM714081 GSM714082 GSM714083 GSM714084 GSM714085

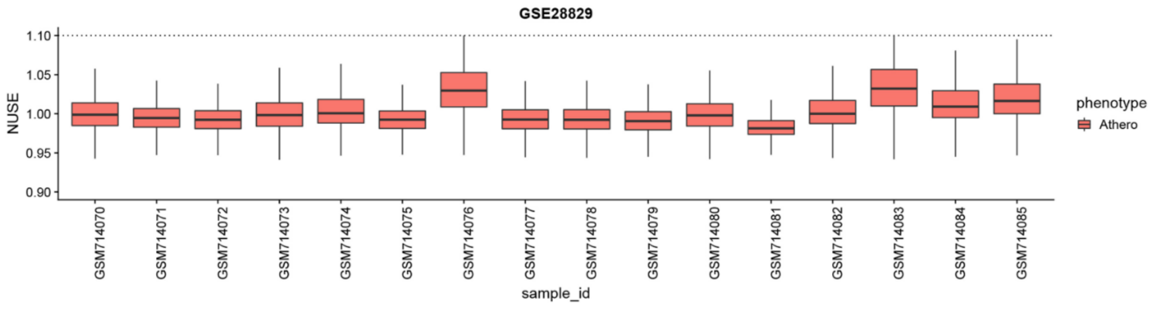
GSE28829
NUSE
sample_id
phenotype
Athero
GSM714070 GSM714071 GSM714072 GSM714073 GSM714074 GSM714075 GSM714076 GSM714077 GSM714078 GSM714079 GSM714080 GSM714081 GSM714082 GSM714083 GSM714084 GSM714085

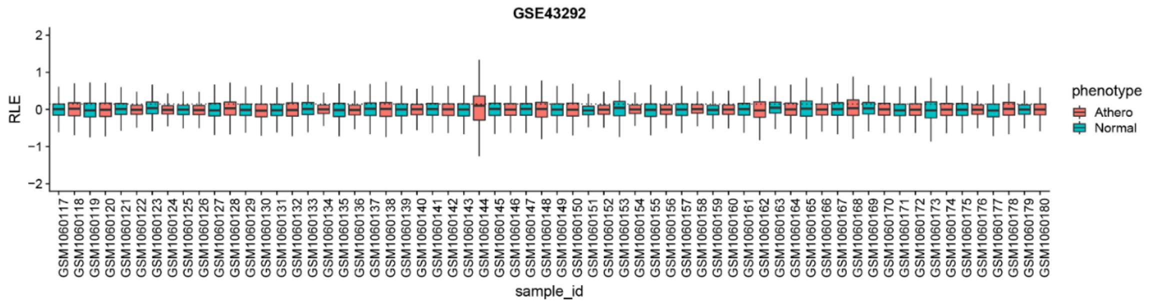
GSE43292
RLE
sample_id
phenotype
Athero
Normal
GSM1060117 … GSM1060180

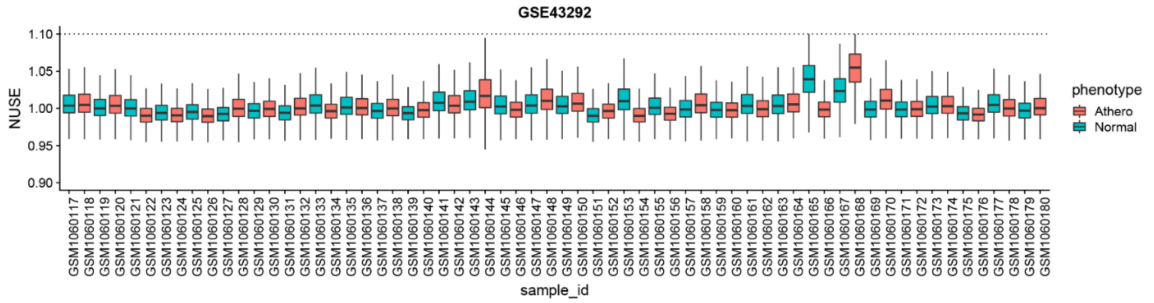
GSE43292
NUSE
sample_id
phenotype
Athero
Normal
GSM1060117 … GSM1060180

Figure S2

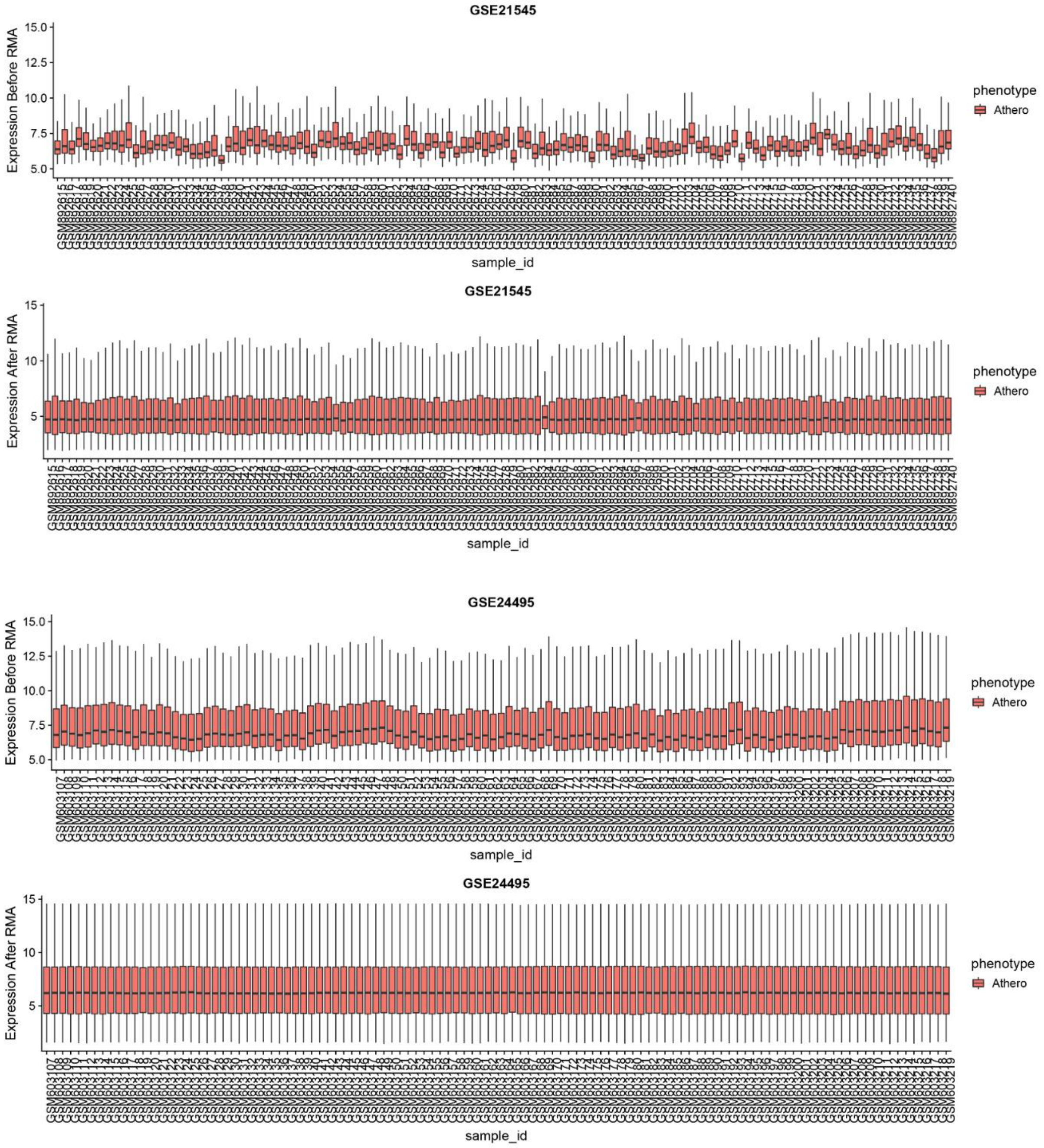
GSE21545
Expression Before RMA
15.0
12.5
10.0
7.5
5.0
sample_id
phenotype
Athero
GSE21545
Expression After RMA
15
10
5
sample_id
phenotype
Athero
GSE24495
Expression Before RMA
15.0
12.5
10.0
7.5
5.0
sample_id
phenotype
Athero
GSE24495
Expression After RMA
15
10
5
sample_id
phenotype
Athero

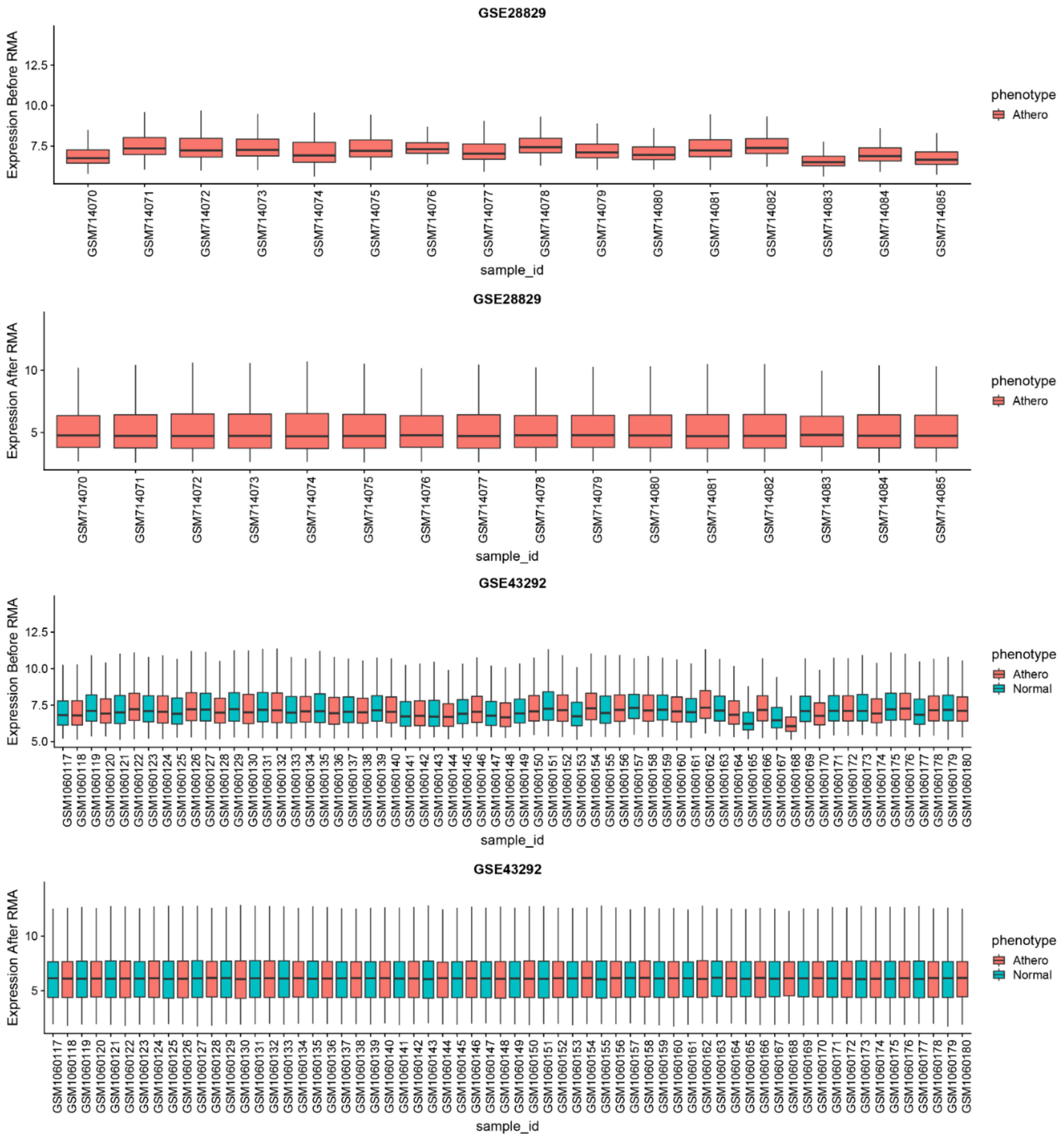
GSE28829
Expression Before RMA
12.5
10.0
7.5
phenotype
Athero
GSM714070
GSM714071
GSM714072
GSM714073
GSM714074
GSM714075
GSM714076
GSM714077
GSM714078
GSM714079
GSM714080
GSM714081
GSM714082
GSM714083
GSM714084
GSM714085
sample_id
GSE28829
Expression After RMA
10
5
phenotype
Athero
GSM714070
GSM714071
GSM714072
GSM714073
GSM714074
GSM714075
GSM714076
GSM714077
GSM714078
GSM714079
GSM714080
GSM714081
GSM714082
GSM714083
GSM714084
GSM714085
sample_id
GSE43292
Expression Before RMA
12.5
10.0
7.5
5.0
phenotype
Athero
Normal
GSM1060117 … GSM1060180
sample_id
GSE43292
Expression After RMA
10
5
phenotype
Athero
Normal
GSM1060117 … GSM1060180
sample_id

Figure S3

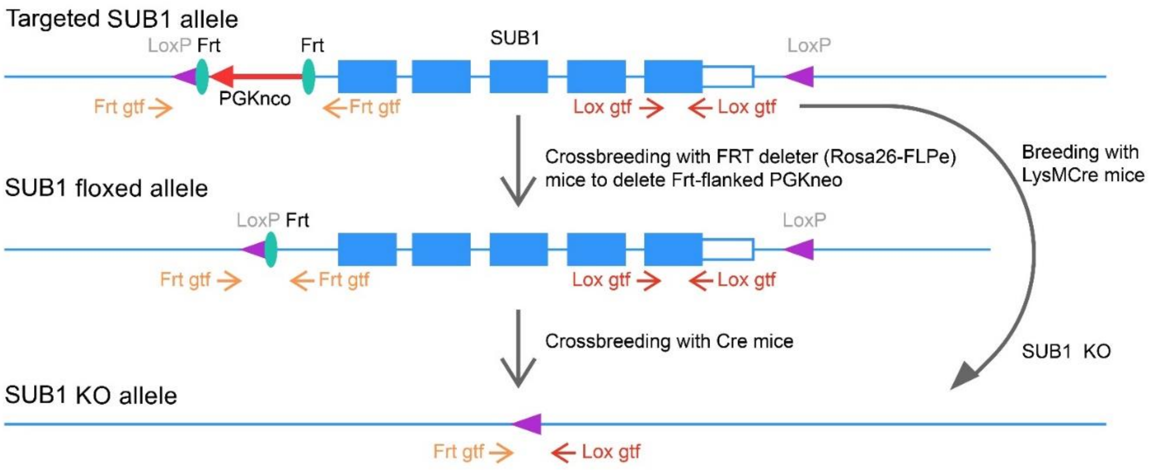
Targeted SUB1 allele
LoxP Frt
Frt
SUB1
LoxP
PGKnco
Frt gtf
Frt gtf
Lox gtf
Lox gtf
Crossbreeding with FRT deleter (Rosa26-FLPe)
mice to delete Frt-flanked PGKneo
Breeding with
LysMCre mice
SUB1 floxed allele
LoxP Frt
LoxP
Frt gtf
Frt gtf
Lox gtf
Lox gtf
Crossbreeding with Cre mice
SUB1 KO
SUB1 KO allele
Frt gtf
Lox gtf

Figure S4

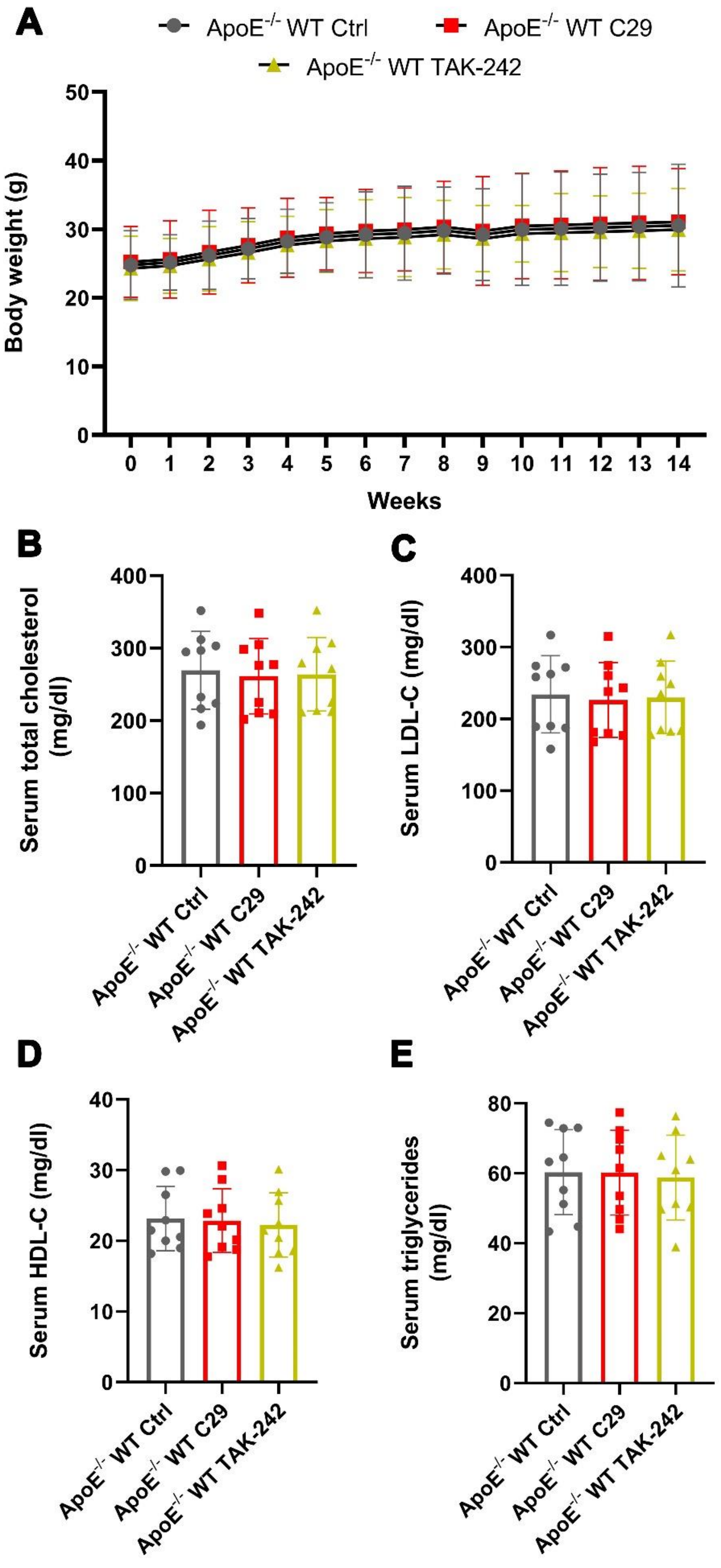
A
ApoE-/- WT Ctrl
ApoE-/- WT C29
ApoE-/- WT TAK-242
Body weight (g)
Weeks
0 1 2 3 4 5 6 7 8 9 10 11 12 13 14
0 10 20 30 40 50
B
Serum total cholesterol (mg/dl)
0 100 200 300 400
C
Serum LDL-C (mg/dl)
D
Serum HDL-C (mg/dl)
0 10 20 30 40
E
Serum triglycerides (mg/dl)
0 20 40 60 80
ApoE-/- WT Ctrl
ApoE-/- WT C29
ApoE-/- WT TAK-242

Figure S5

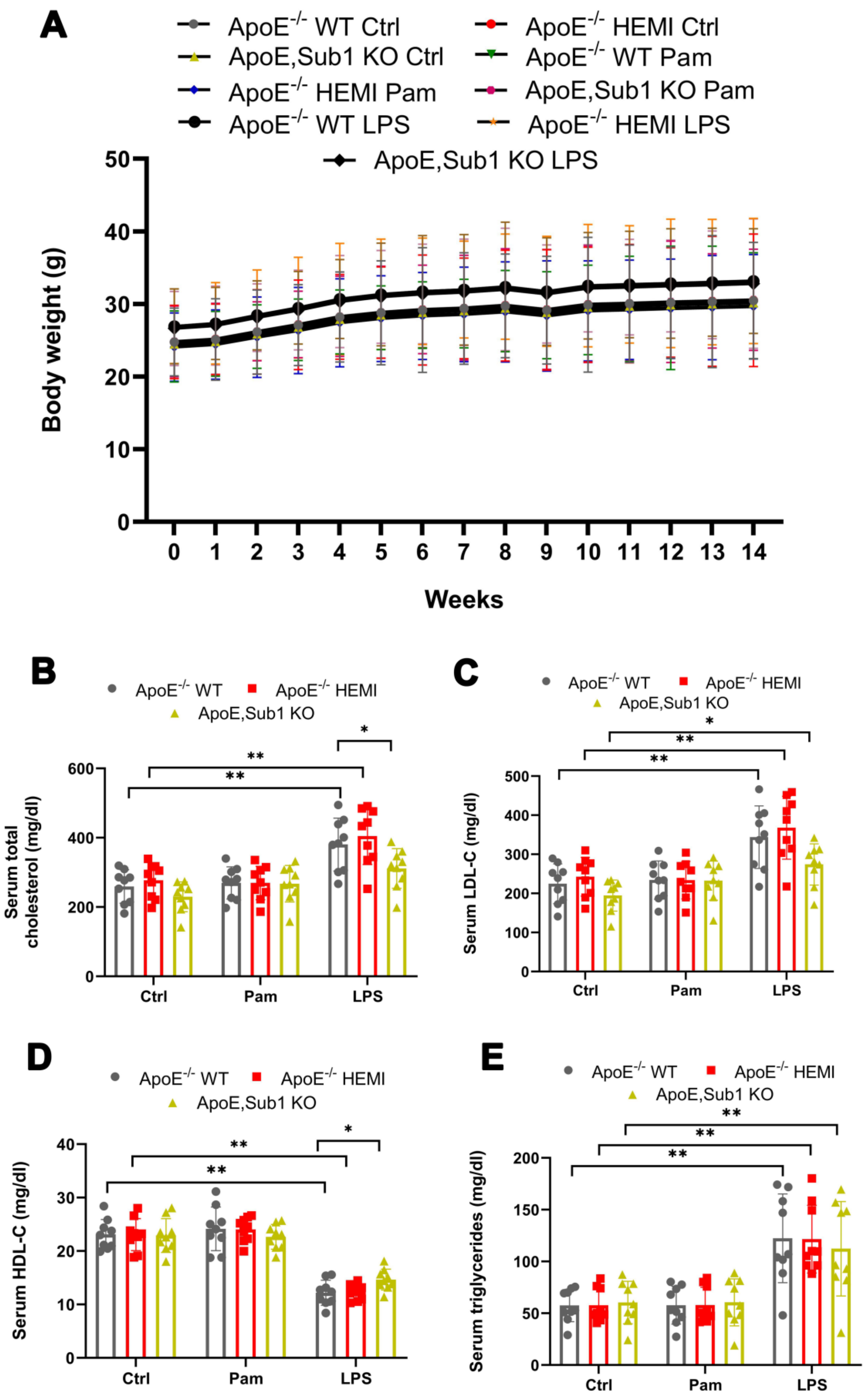
A
ApoE-/- WT Ctrl
ApoE-/- HEMI Ctrl
ApoE,Sub1 KO Ctrl
ApoE-/- WT Pam
ApoE-/- HEMI Pam
ApoE,Sub1 KO Pam
ApoE-/- WT LPS
ApoE-/- HEMI LPS
ApoE,Sub1 KO LPS
Body weight (g)
Weeks
B
ApoE-/- WT
ApoE-/- HEMI
ApoE,Sub1 KO
Serum total cholesterol (mg/dl)
Ctrl
Pam
LPS
C
Serum LDL-C (mg/dl)
D
Serum HDL-C (mg/dl)
E
Serum triglycerides (mg/dl)

Figure S6

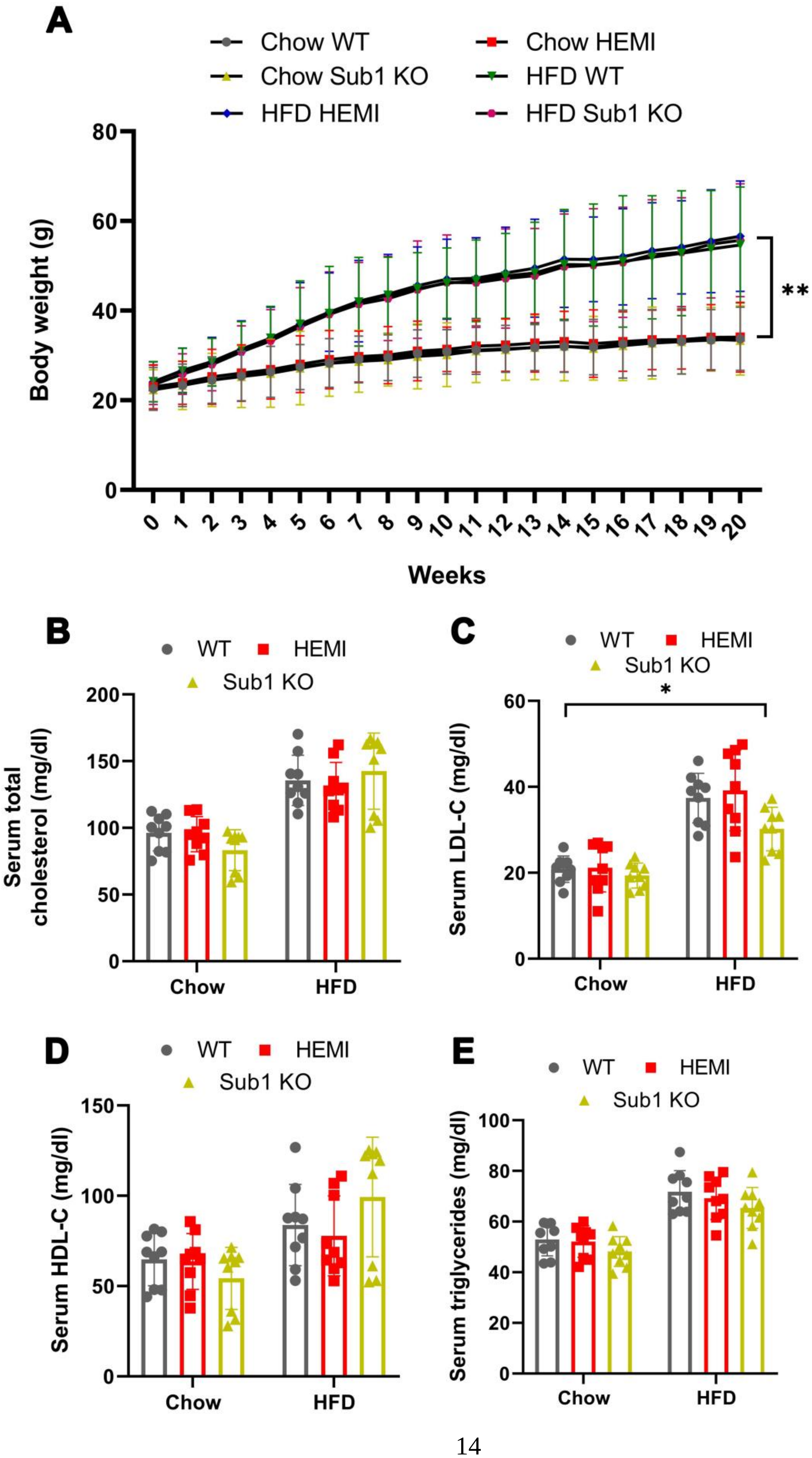
A
Chow WT
Chow HEMI
Chow Sub1 KO
HFD WT
HFD HEMI
HFD Sub1 KO
Body weight (g)
Weeks
**
B
WT
HEMI
Sub1 KO
Serum total cholesterol (mg/dl)
Chow
HFD
C
Serum LDL-C (mg/dl)
*
D
Serum HDL-C (mg/dl)
E
Serum triglycerides (mg/dl)

Figure S7

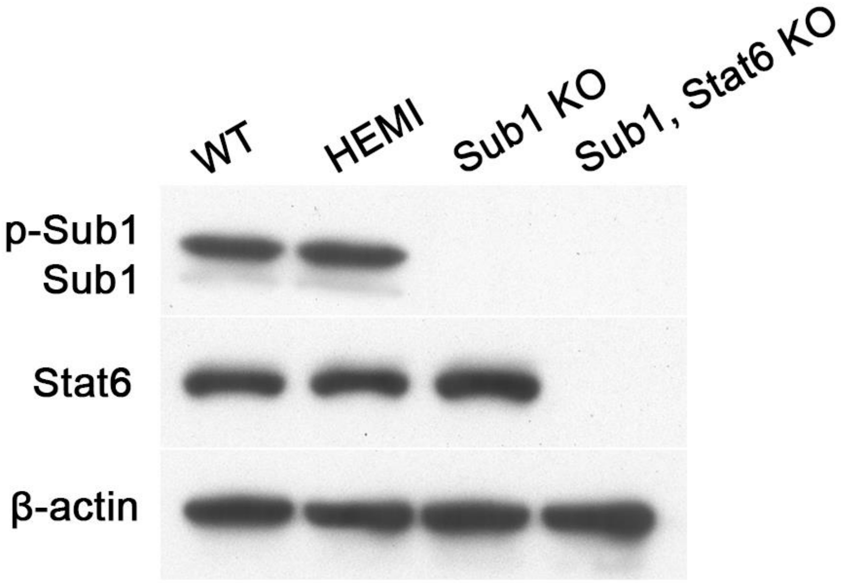

Figure S8

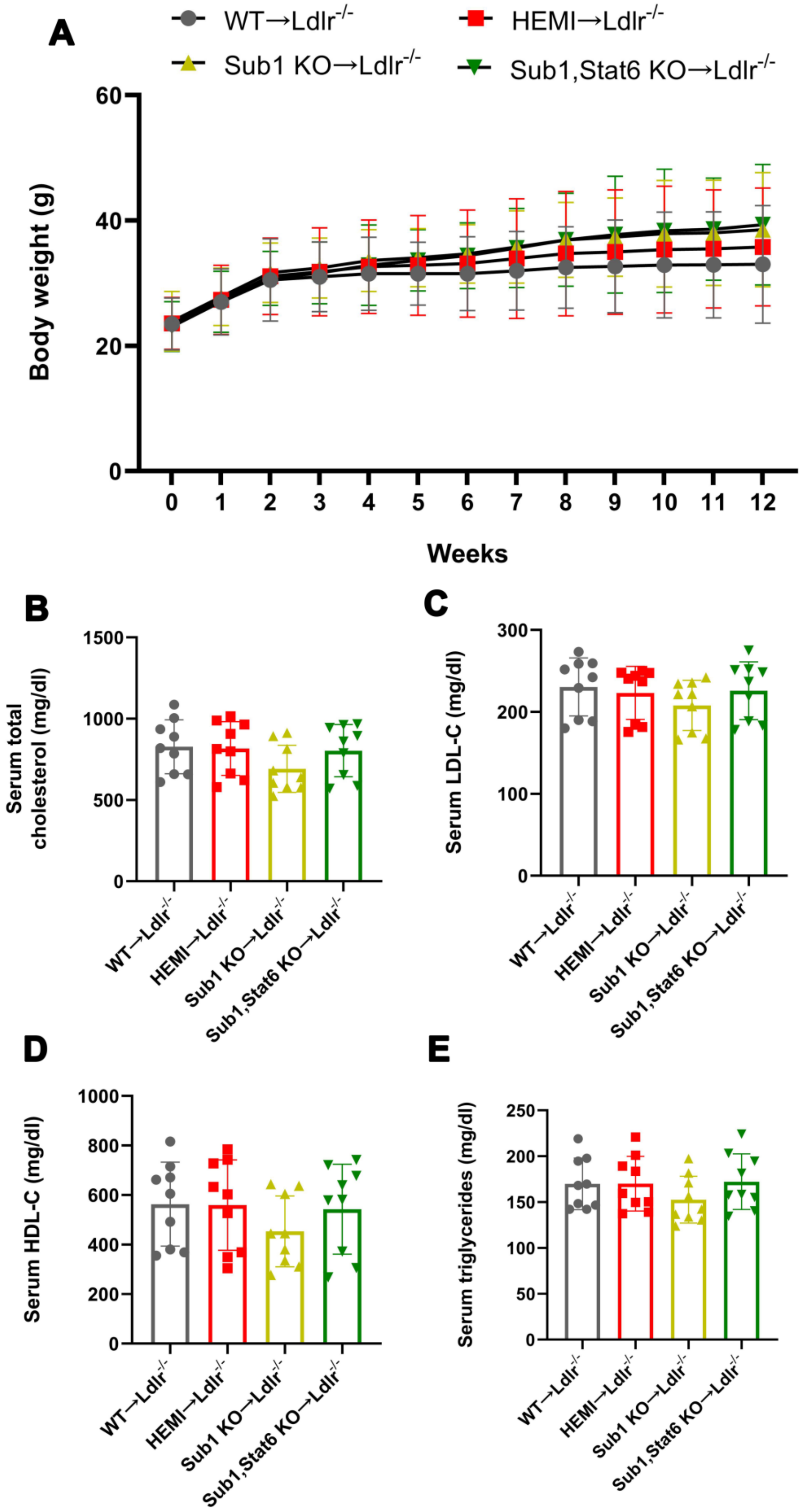
A
WT→Ldlr-/-
HEMI→Ldlr-/-
Sub1 KO→Ldlr-/-
Sub1,Stat6 KO→Ldlr-/-
Body weight (g)
Weeks
B
Serum total cholesterol (mg/dl)
C
Serum LDL-C (mg/dl)
D
Serum HDL-C (mg/dl)
E
Serum triglycerides (mg/dl)

Figure S9

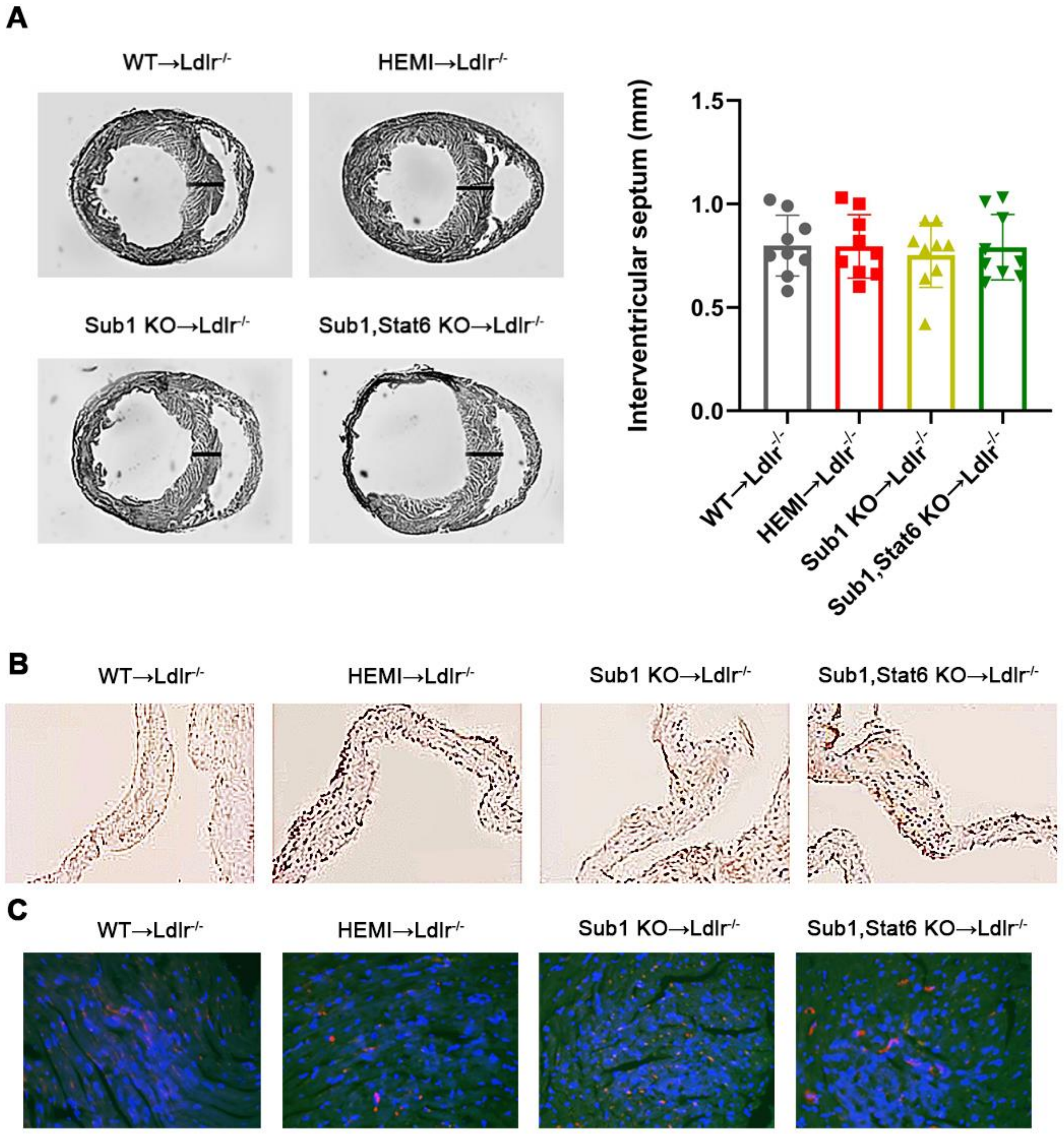
A
WT→Ldlr-/-
HEMI→Ldlr-/-
Sub1 KO→Ldlr-/-
Sub1,Stat6 KO→Ldlr-/-
Interventricular septum (mm)
1.5
1.0
0.5
0.0
WT→Ldlr-/-
HEMI→Ldlr-/-
Sub1 KO→Ldlr-/-
Sub1,Stat6 KO→Ldlr-/-
B
WT→Ldlr-/-
HEMI→Ldlr-/-
Sub1 KO→Ldlr-/-
Sub1,Stat6 KO→Ldlr-/-
C
WT→Ldlr-/-
HEMI→Ldlr-/-
Sub1 KO→Ldlr-/-
Sub1,Stat6 KO→Ldlr-/-

Figure S10

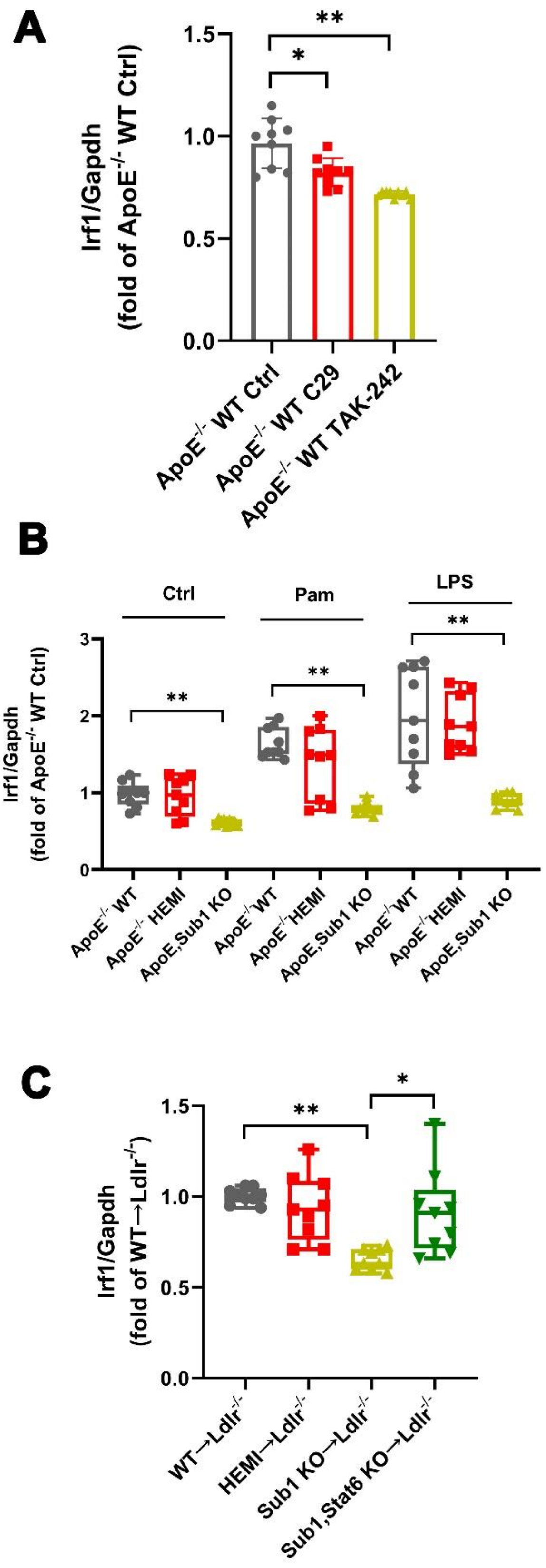
A
Irf1/Gapdh
(fold of ApoE-/- WT Ctrl)
1.5
1.0
0.5
0.0
**
*
ApoE-/- WT Ctrl
ApoE-/- WT C29
ApoE-/- WT TAK-242
B
Ctrl
Pam
LPS
Irf1/Gapdh
(fold of ApoE-/- WT Ctrl)
3
2
1
0
**
**
**
ApoE-/- WT
ApoE-/- HEMI
ApoE,Sub1 KO
ApoE-/-WT
ApoE-/-HEMI
ApoE,Sub1 KO
ApoE-/-WT
ApoE-/-HEMI
ApoE,Sub1 KO
C
Irf1/Gapdh
(fold of WT→Ldlr-/-)
1.5
1.0
0.5
0.0
**
*
WT→Ldlr-/-
HEMI→Ldlr-/-
Sub1 KO→Ldlr-/-
Sub1,Stat6 KO→Ldlr-/-

Figure S11

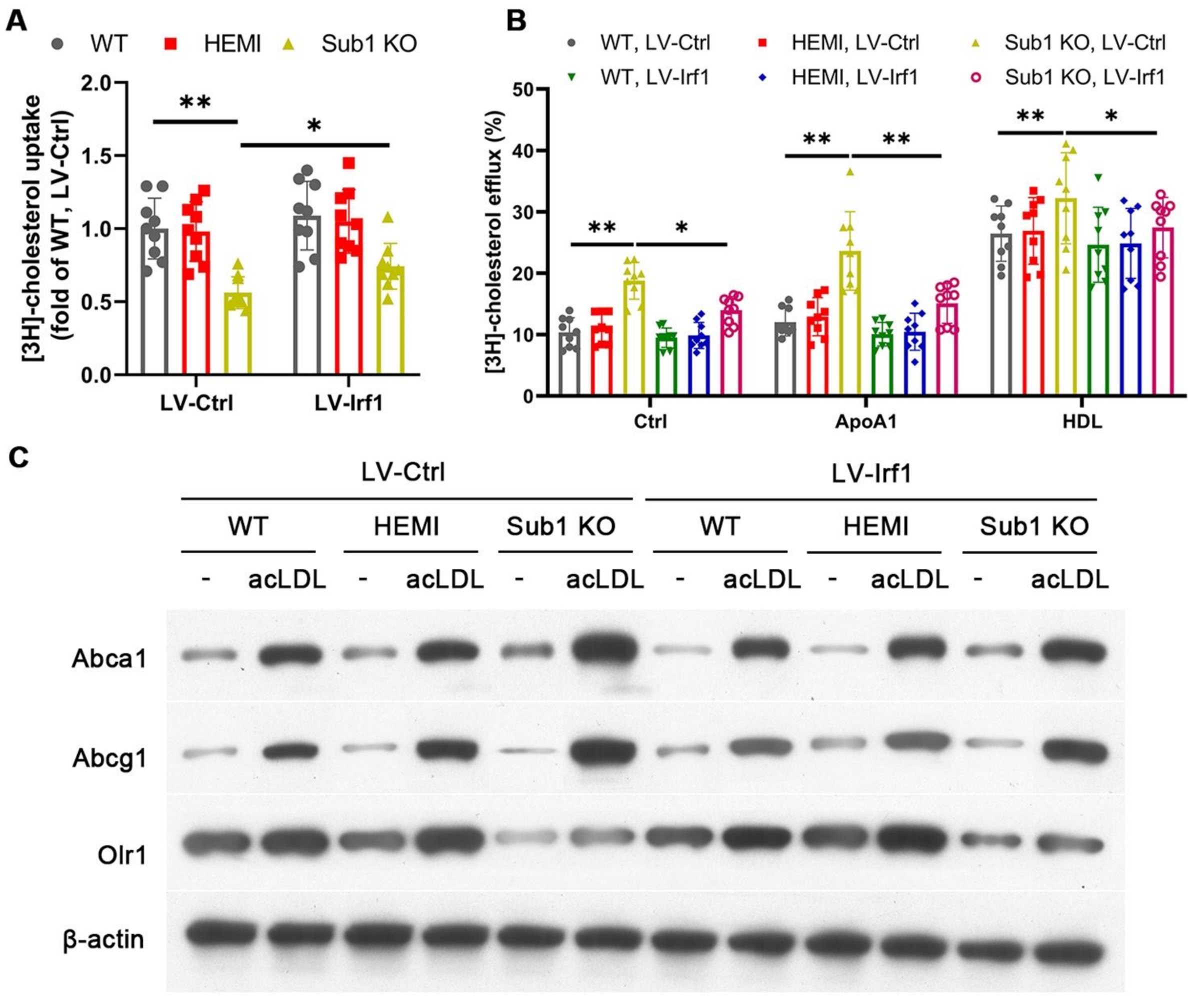
A
WT
HEMI
Sub1 KO
[3H]-cholesterol uptake (fold of WT, LV-Ctrl)
2.0
1.5
1.0
0.5
0.0
**
*
LV-Ctrl
LV-Irf1
B
WT, LV-Ctrl
HEMI, LV-Ctrl
Sub1 KO, LV-Ctrl
WT, LV-Irf1
HEMI, LV-Irf1
Sub1 KO, LV-Irf1
[3H]-cholesterol efflux (%)
50
40
30
20
10
0
Ctrl
ApoA1
HDL
C
LV-Ctrl
LV-Irf1
WT
HEMI
Sub1 KO
-
acLDL
Abca1
Abcg1
Olr1
β-actin
